# Functionalization of curcumin nanomedicines: a recent promising adaptation to maximize pharmacokinetic profile, specific cell internalization and anticancer efficacy against breast cancer

**DOI:** 10.1186/s12951-023-01854-x

**Published:** 2023-03-25

**Authors:** Jinku Zhang, Jirui Sun, Chong Li, Haizhi Qiao, Zahid Hussain

**Affiliations:** 1 Department of Pathology, Baoding First Central Hospital, Baoding, 071000 Hebei China; 2 Core Facility for Protein Research, Institute of Biophysics, Chinese Academy of Sciences, Beijing, 100101 China; 3 Department of Pharmaceutics and Pharmaceutical Technology, College of Pharmacy, University of Sharjah, 27272 Sharjah, United Arab Emirates; 4 Research Institute for Medical and Health Sciences, University of Sharjah, 27272 Sharjah, United Arab Emirates

**Keywords:** Curcumin nanomedicines, Functionalization, Pharmacokinetics, Passive and selective targeting, Anticancer efficacy, Breast cancer

## Abstract

Owing to its diverse heterogeneity, aggressive nature, enormous metastatic potential, and high remission rate, the breast cancer (BC) is among the most prevalent types of cancer associated with high mortality. Curcumin (Cur) is a potent phytoconstituent that has gained remarkable recognition due to exceptional biomedical viability against a wide range of ailments including the BC. Despite exhibiting a strong anticancer potential, the clinical translation of Cur is restricted due to intrinsic physicochemical properties such as low aqueous solubility, chemical instability, low bioavailability, and short plasma half-life. To overcome these shortcomings, nanotechnology-aided developments have been extensively deployed. The implication of nanotechnology has pointedly improved the physicochemical properties, pharmacokinetic profile, cell internalization, and anticancer efficacy of Cur; however, majority of Cur-nanomedicines are still facing grandeur challenges. The advent of various functionalization strategies such as PEGylation, surface decoration with different moieties, stimuli-responsiveness (i.e., pH, light, temperature, heat, etc.), tethering of specific targeting ligand(s) based on the biochemical targets (e.g., folic acid receptors, transferrin receptors, CD44, etc.), and multifunctionalization (multiple functionalities) has revolutionized the fate of Cur-nanomedicines. This study ponders the biomedical significance of various Cur-nanomedicines and adaptable functionalizations for amplifying the physicochemical properties, cytotoxicity via induction of apoptosis, cell internalization, bioavailability, passive and active targeting to the tumor microenvironment (TME), and anticancer efficacy of the Cur while reversing the multidrug resistance (MDR) and reoccurrence in BC. Nevertheless, the therapeutic outcomes of Cur-nanomedicines against the BC have been remarkably improved after adaptation of various functionalizations; however, this evolving strategy still demands extensive research for scalable clinical translation.

**Graphical Abstract:**

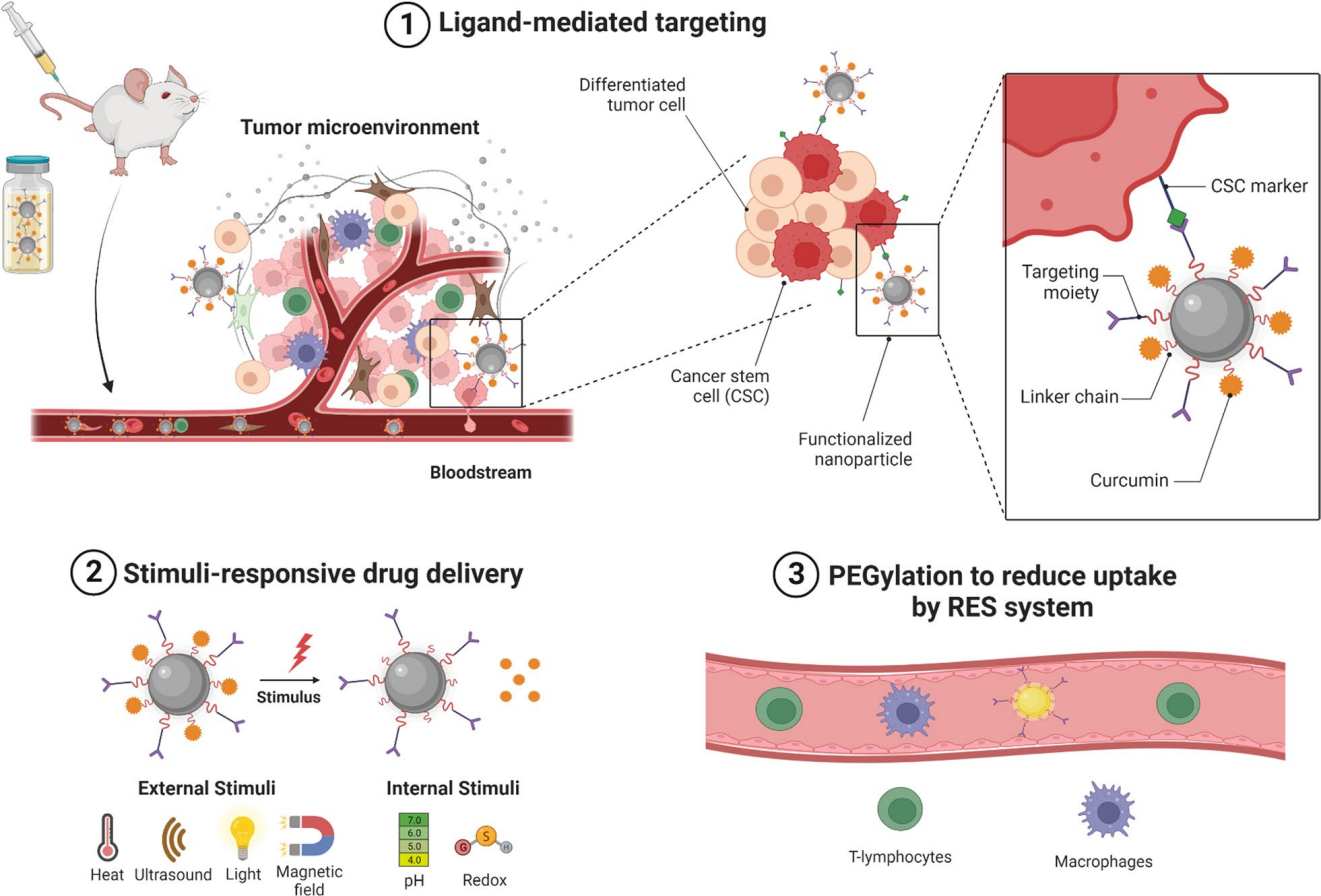

## Introduction

### Breast cancer: prevalence, signs and symptoms

Owing to its aggressive nature, heterogeneity, and immense potential to metastasize to other body organs (i.e., lung, liver, bone, brain, skin, etc.), breast cancer (BC) is among the most prevalent types of cancer that occurs in women (also in men). According to an estimate, approximately 458,000 deaths and more than one million new cases of BC happen each year globally [1, 2]. According to the American cancer society, the most common type of cancer diagnosed in the American women between 2019 and 2021 was BC with an increased incidence rate of 0.5% per year and after the lung carcinoma it was second most prominent cause of mortality in women. Similarly, according to a recent statistical estimation of International Agency for Research on Cancer (IARC) which is a part of World Health Organization (WHO) of United Nation (UN), BC was the first among the five most diagnosed types of cancer in UAE with 1030 new cases in 2020 [3, 4]. Various signs and symptoms of BC include obvious changes in the size and/or shape of the nipple or breast, presence of lump(s) or swelling in the breast, severe consistent pain in the breast, nipple area or underarm (armpits), discharge of blood from the nipple, and redness or flaky areas around the nipple or on the breast [5]. If one or more than one such signs or symptoms appear in a patient, it is highly recommended to immediately perform certain tests including the breast ultrasound, mammogram, magnetic resonance imaging (MRI), and/or breast biopsy for further investigation and diagnosis.

It has been established that the overall survival rate and diseases free survival are higher when BC is diagnosed at early stages or treated with rational therapeutic regimen [4, 5]. Detection of BC at early stages leads to better therapeutic outcome: 5-year survival rate is 100% when BC diagnosed at stage I in comparison with only 20% survival rate when BC diagnosed at advanced stages (stages III and IV) [6, 7].

### Staging

**Fig. 1:**
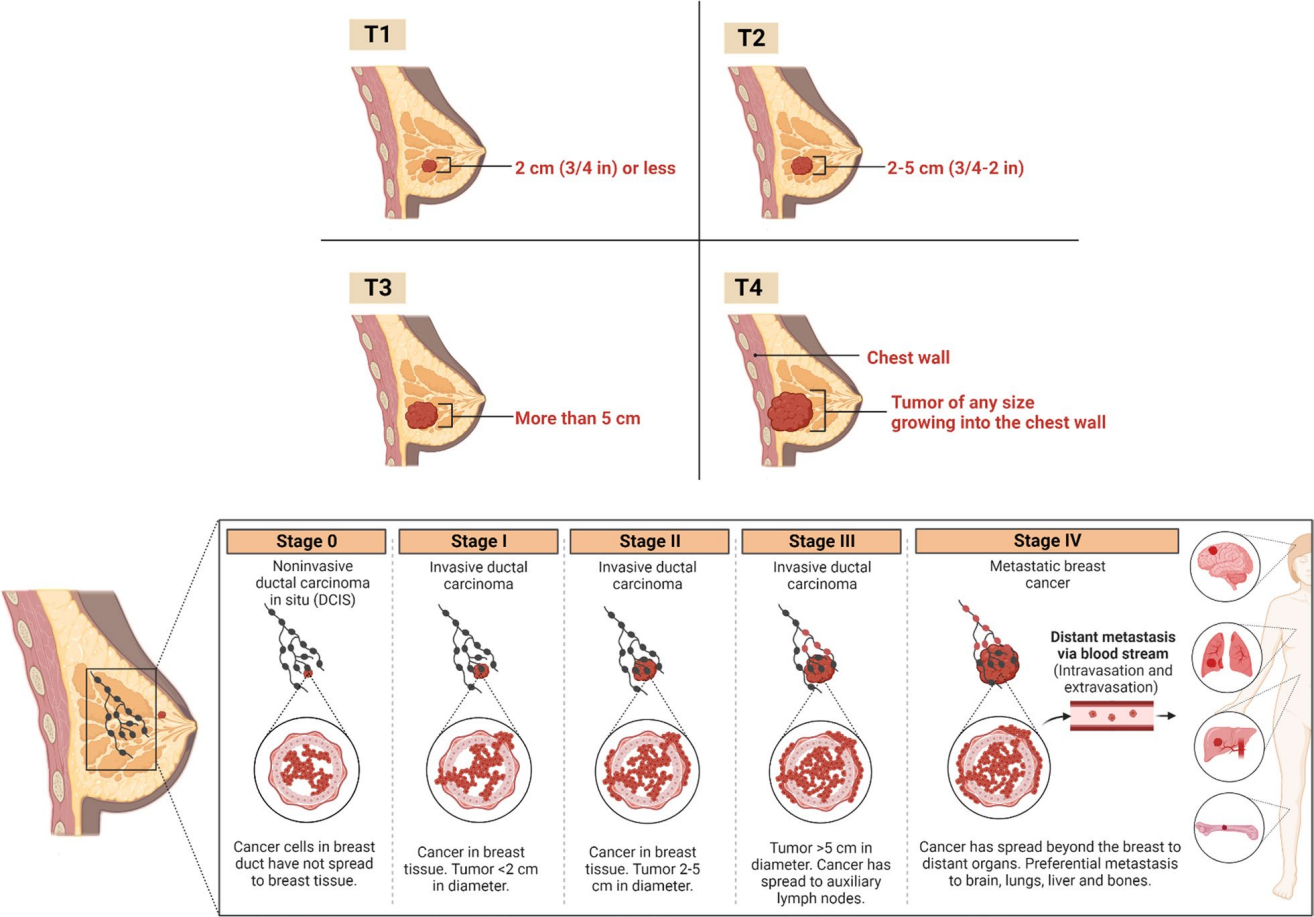
Stages T1–T4 describe the size of tumor and extent of metastasis to the chest wall (local metastasis) or to other organs of the body (distant metastasis). Created with BioRender.com

For rationalizing a most suitable therapeutic regimen for BC patient, staging is critically important. Staging also helps to; (1) assess the therapeutic success of prescribed medication and results of clinical trials, (2) evaluate the survival statistics, (3) exchange or compare the medical information between the various treatment centers, and (4) serves as a baseline for translational research. For staging of BC, TNM system (Tumor, Lymph node, Metastasis) of American Joint Committee on Cancer (AJCC) is most employed (Table 1). The stage of the BC is usually determined from characteristics of the cancer such as how big is the lump/tumor, extent of metastasis (local or distant), and involvement of hormone receptors (i.e., estrogen, progesterone, and HER2 status) (Fig. 1) [4].

**Table 1:** Staging of BC using TNM staging system

Tumor size (T)	Tumor can’t be assessed TX	No tumor T0	Tumor size < 2 cm T1	Tumor size 2–5 cm T2	Tumor size > 5 cm T3	Tumor extends to skin or chest wall T4
Lymph nodes (N)	lymph nodes can't be assessed NX	No lymph node metastasis N0	Metastasis to ipsilateral, movable, axillary lymph nodes N1	Metastasis to ipsilateral fixed axillary, or IM lymph nodes N2	Metastasis to infraclavicular/ supraclavicular lymph nodes, or to axillary and IM lymph nodes N3	—
Metastasis (M)	metastasis can't be assessed MX	No distant metastasis M0	Distant metastasis M1	—	—	—

Stage I: (T1, N0, M0).

Stage II (A, B).

IIA: 1) T0, N1, M0; 2) T1, N1, M0; 3) T2, N0, M0.

IIB: 1) T2, N1, M0; 2) T3, N0, M0; 3) T3, N1, M0.

Stage III (A, B, C).

IIIA: 1) T0, N2, M0; 2) T1, N2, M0; 3) T2, N2, M0; 4) T3, N2, M0; 5) T3, N1, M0.

IIIB: 1) T4, N0, M0; 2) T4, N1, M0; 3) T4, N2, M0.

IIIC: 1) Any T, N3, M0.

Stage IV: Any T, any N, M1.

### Pathophysiology and risk factors

Owing to its intricate and multi-factorial nature, pathophysiology of BC is not fully understood yet; however, probability of developing BC could be enhanced due to certain risk factors. For example, old age is one of the major risk factors for developing the BC. Females aged below 25 rarely develop BC; however, women with age 50–69 have higher probability of developing BC [8, 9]. Other risk factors include genetic mutation (e.g., BRCA 1 and 2) that accounts for about 10% of BC cases [10], induction of P53, overexpression of cyclin D gene [11], higher body mass index (BMI), late pregnancy (> 30 years) [12], family history of BC or other non-cancerous breast diseases, any previous treatments involving radiations, late menopause, and postmenopausal hormone replacement therapy (HRT) [13].

### Metastasis to other organs

The spreading of BC from the primary site (i.e., breast duct) to the surrounding healthy cells/tissues (local metastasis) or to other organs of the body (e.g., bones, brain, lungs, liver, etc.) (distant metastasis) is called metastasis. Following its metastasis, it is still considered as BC because the cells (also called as circulating tumor cells) which metastasize to other organs are BC cells which broke away from the original tumor and invaded to nearby tissues or travelled through the bloodstream or lymph nodes to the distant organs. Stage IV BC, also called as metastatic BC, is characterized by distinct spreading to other organs and thus the overall survival rate in these patients is usually below 20%. Even months after the successful treatment of BC, it can still reappear in other organs which is called metastatic reoccurrence or distant reoccurrence.

**Fig. 2:**
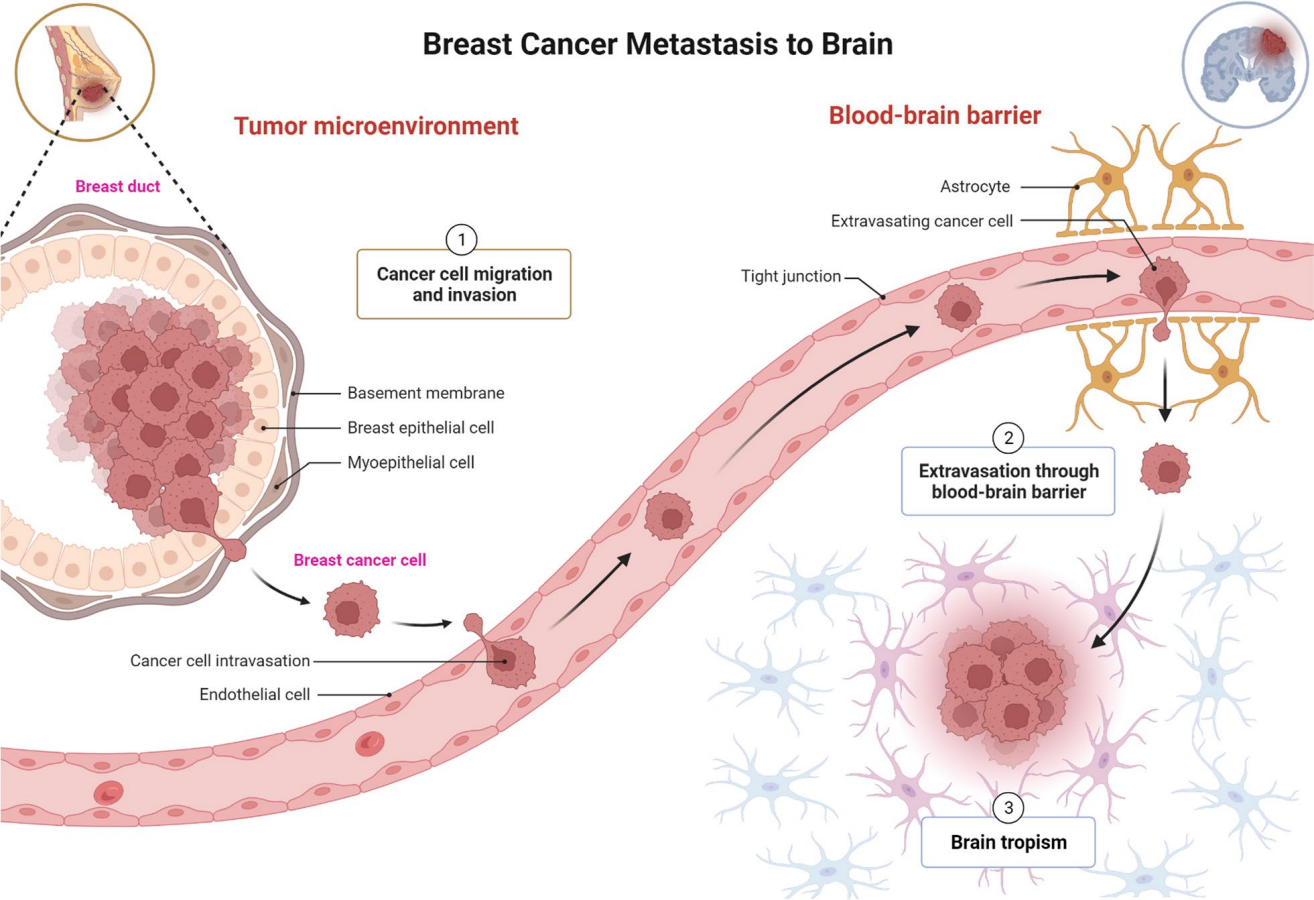
BC metastasis to brain tissues (distant metastasis). Created with BioRender.com

Metastasis is a complex interplay involving the multiple cellular processes including the hyperproliferation (cell division) of primary tumor cells, cell invasion through the basement membrane to the surrounding tissues, cell intravasation to the bloodstream or lymphatic system, cell migration through blood circulation or lymph, extravasation from the bloodstream via trans-endothelial migration, invasion into the distant organs, multiplication or cell division into distant tissue, and formation of distant tumor (Fig. 2). The organs that are commonly metastasized with BC include the brain, liver, bones, and lungs. Over 10–30% of patients with metastatic BC contain BC cells invaded into the brain tissues. Symptoms of metastatic BC depends upon the organ to which it has been metastasized. For example, severe progressive generalized pain, profound fatigue, and easily fractured bone are the typical symptoms of BC metastasized to bone tissues. Progressive headache, dizziness, vomiting, nausea, seizures, and visual disturbance are typical signs of BC metastasized to the brain. Abdominal pain, nausea, vomiting, stomach swelling, increase in the liver enzymes, and jaundice indicates metastasis of BC to the liver.

## Conventional treatments for BC and limitations

There are three most exploited conventional strategies for the management of BC such as chemotherapy, surgery, and radiation therapy; however, other techniques can also be used in some cases such as personalized medicine, immunotherapy, hormonal therapy, and bone marrow transplant.

### Chemotherapy

Chemotherapy involves the administration of anticancer drugs that can effectively target and destroy the BC cells with minimal toxicity to the surrounding healthy cells. Anticancer drugs are frequently administered intravenously (injection) or through oral route (pills or tablets). Depending upon the patient condition and the cancer stage, chemotherapy is administered alone or in combination with other therapies such as radiation, surgery and/or hormonal therapy. Chemotherapeutic drugs help relieving the symptoms, mitigating the spread, and preventing the reoccurrence of BC. As an adjuvant, chemotherapeutic agents can also be given to BC patients after undergoing the surgical intervention for complete eradication of the remaining cancerous cells as well as preventing the reoccurrence of the disease [14]. Chemotherapeutic agents can also be administered before carrying out the surgery in BC patients in order to shrink the tumor size thus decreasing the severity and extent of surgical excision. This therapy is called neo-adjuvant chemotherapy or preoperative chemotherapy. Neo-adjuvant chemotherapy is usually recommended in HER2 positive BC, triple negative BC, inflammatory BC, BC metastasized to lymph nodes, and all advanced stage BC (stages III and IV). In advanced stage BC, chemotherapy is the first-choice treatment modality because BC has metastasized to other organs (e.g., lungs, liver, bone, brain, etc.) of the body and thus surgery is not the viable option in these situations. Often a combined chemotherapeutic regimen involving the multiple chemotherapeutic agents are recommended for such patients to improve their quality of life, disease free survival, and overall survival rates [15].

According to EBCTCG studies, adjuvant or neo-adjuvant chemotherapy or a combined chemotherapy significantly decrease the mortality rate and probability of recurrence of BC, particularly in patients with age less than 50. Administration of cyclophosphamide with methotrexate and 5-fluorouracil shows major effectiveness in node-positive tumors in premenopausal patients. The use of anthracyclines has shown good efficacy as combination chemotherapy regardless of menopausal status as well as in ER-positive tumors [16]. Another chemotherapeutic agent which has shown good efficacy against ER-positive tumor is tamoxifen; however, according to some clinical trials, use of chemo-endocrine therapy showed more significant effects compared to tamoxifen alone. A common regimen practiced in the United States is the combination of doxorubicin and cyclophosphamide as four cycles, followed by four cycles with paclitaxel. The dose–dense (dd) AC-T is administered along with growth factor every 2 weeks [17]. According to a meta-analysis, there is a significant benefit of chemotherapy for patients with HR-negative BC in decreasing the recurrence and mortality [18].

Chemotherapeutic agents are also commonly recommended as systemic adjuvant therapy to BC patients after the surgical resection to eradicate micro-metastatic tumor that might progress at later stages (if not treated properly). The criterion of the selection of an adjuvant therapy is based on BC burden including the number of lymph nodes involved, primary tumor size and the pathophysiology involved. Patients with triple negative and HER2 positive cancers are at higher risk, although in HR positive and HER2 negative cancers there is a biological diversity. According to a clinical trial, HR positive, HER2 negative, and node-negative BC cases have shown good response to chemotherapy as an adjuvant therapy [19].

Another standard regimen is docetaxel with AC (DAC), although there is an increased risk of toxicity and febrile neutropenia with docetaxel use [24]. According to the trial data from Cancer and Leukemia Group B and US Breast Cancer Intergroup, chemotherapy shows decrease in the relative risk in HR negative cancer patients about 21–25%, and HR positive cancer of 8–12% relative risk reduction. Oncotype DX gives an estimation about the benefits of chemotherapy where higher oncotype recurrence above 31 shows greater risk reduction of recurrence with chemotherapy [20].

HER2 targeted therapy can also be given in combination with chemotherapeutic agent to HER2 positive BC patients. Results of this randomized trial indicated that trastuzumab combined with chemotherapy against HER2 receptor BC patients have shown 50% reduction in the recurrence rate [21]. Similarly, trastuzumab has been given in combination with paclitaxel to stage I HER2 positive BC patients, while stage II-III HER2 positive BC patients were given trastuzumab with AC-T or docetaxel and carboplatin. Pertuzumab which is a HER2 dimerization inhibitor has also shown good anti-BC response when administered in combination with trastuzumab [22].

### Limitations of chemotherapy

Though, chemotherapy is considered as a first-choice therapy for advanced stage BC, either alone or in combination with other therapeutic modalities; however, several limitations including poor selectivity which results in promising cytotoxicity in normal healthy cells having high proliferation rate limits its clinical significance. Another limitation associated with chemotherapy is “multidrug resistance (MDR)”. One of the mechanisms of MDR is overexpression of efflux pump (P-glycoprotein) which results in efflux (pumping-out) of the internalized anticancer drug and thus results in decreased intracellular levels of anticancer drugs. Another limitation is low aqueous solubility as many chemotherapeutic agents derived from plant or synthetic sources are hydrophobic in nature [23, 24]. Chemotherapy also cause several adverse effects such as alopecia, cognitive and sexual dysfunction, persistent nausea and vomiting, amenorrhea, menstrual pain, and bone pain that affects patient’s quality of life [25]. These limitations reduce therapeutic significance and patient compliance with chemotherapy.

### Surgery

**Fig. 3:**
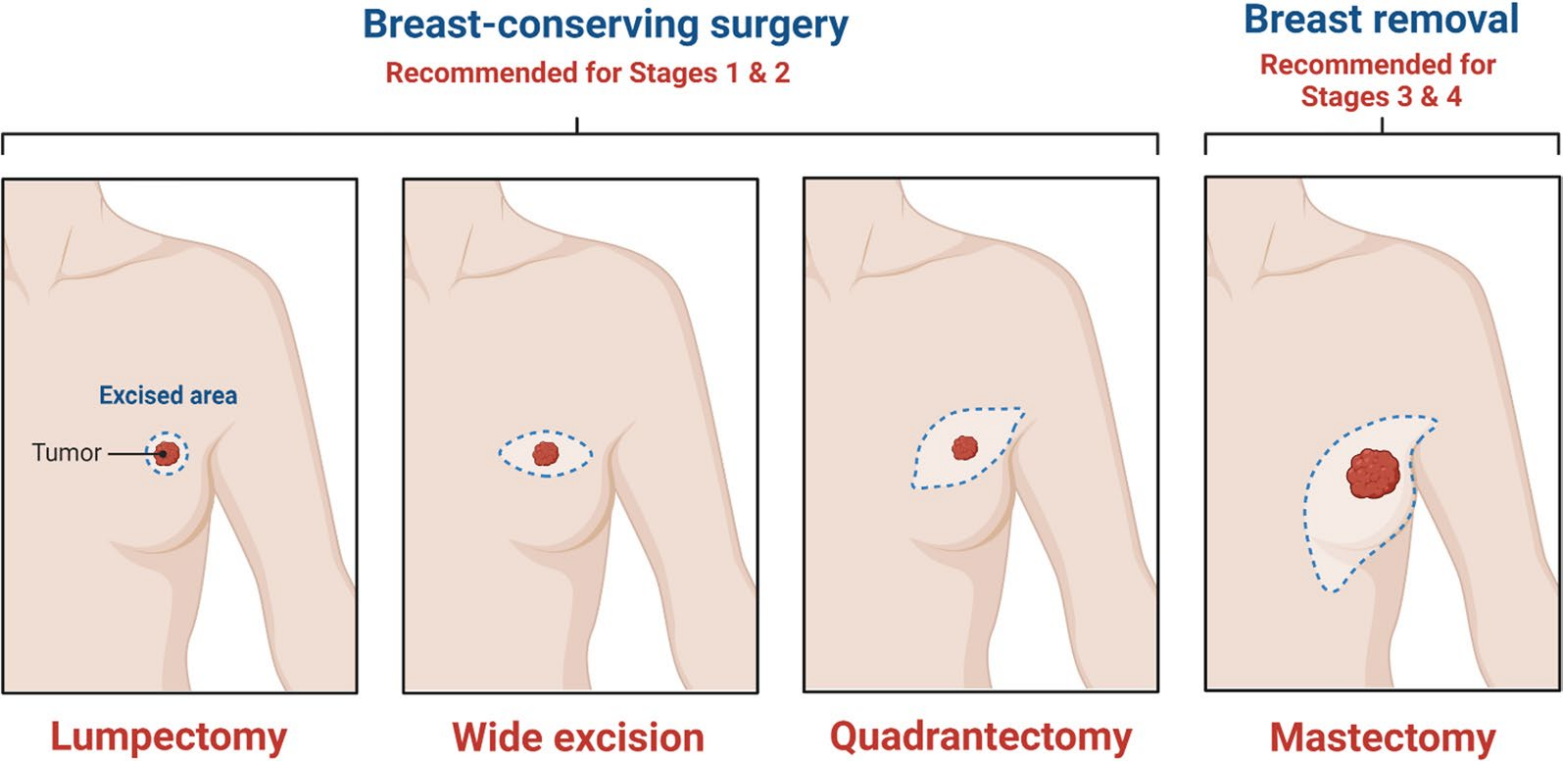
Surgical approaches for treatment of BC. Created with BioRender.com

Surgery is the most adaptable treatment modality for the removal of localized tumors [26]; however, for better therapeutic outcomes, surgery can be adjuvant with radiation or chemotherapy prior to undergoing surgical procedure to shrink the tumor size. The adjuvant therapy can also be recommended after the patient has undergone the surgical procedure to mitigate the risks of reoccurrence and to kill remnants of cancerous tissues. Two main surgical approaches are usually adopted: first is called as breast-conserving surgery in which only the cancerous part of the breast (lumpectomy) or the cancerous part along with the rim of nearby healthy tissues (wide excision) are surgically removed (Fig. 3). Quadrantectomy, also called as partial mastectomy, is also a type of breast-conserving surgery in which one quarter of the breast tissues is removed along with the muscles of chest wall within 2–3 cm radius of the tumor. This is usually an out-patient surgery which takes about 1–2 h and patient can be discharged after the surgery; however, surgeons should take into consideration several factors such as breast size, tumor size, underlying health condition, BC staging, and extent of metastasis [27]. Lumpectomy is normally recommended for patients with early stages (stage I or II). The second surgical approach is mastectomy which involves a complete removal of the breast and can be done on both sides. Generally, it is recommended for stages III and IV patients where tumor size is greater than 5 cm size. Sometime, mastectomy is recommended in case of BC recurrence after a patient has undergone breast-conserving surgery [28].

### Limitations of surgical approach

After undergoing surgical procedure particularly in case of breast conserving surgery, radiation therapy is highly recommended to minimize the risks of recurrence; however, patient must be a good candidate for the radiation therapy otherwise it may result in severe adverse events on follow-up. Moreover, tumor tissue should be excised to negative margins and should obtain good cosmetic results. Other limitations of the surgical approach include formation of calcification in the breast with malignancy features, inability to remove tumor within negative margins, high risk of recurrence, and invasiveness (permanent removal of whole breast with additional reconstructive surgeries particularly after the mastectomy) [27, 28]. These limitations reduce the clinical acceptability of surgical invention for the treatment of BC.

### Radiation therapy

Radiation therapy involves the application of high doses of ionizing radiations directed to the cancerous tissues/cells. It is often recommended in combination with other conventional treatments. For example, prior to the surgical procedure radiotherapy can decrease the size of the tumor or post-mastectomy radiotherapy is recommended to decrease the risks of recurrence as well as to improve patient’s quality of life and overall survival rates [29]. The ionizing radiations induce deterioration in genetic material of BC cells which ultimately stop their proliferation via arresting the cycle, migration and metastasis and promote their apoptosis. Generally, conventional radiotherapy requires twenty-five to thirty sessions depending upon the patient condition and BC staging. Alternatively, hypofractionation radiation therapy involves lesser number of sessions (thirteen to sixteen) but with higher doses; however, it depends on various patient’s factors such as age and metastatic status as well as the inferior therapeutic outcomes (e.g., low reconstruction or cosmetic benefits) [30].

### Limitations of radiotherapy

Radiation therapy is considered as an essential modality for cancer treatment with substantial significance; however, it is associated with several problems such as damage to non-cancerous surrounding tissues/cells which results in chromosomal abnormalities and structural changes in patients receiving the radiotherapy. Several other side effects have also been reported in BC patients undergone radiotherapy such as skin irritation and dermatitis, heaviness and swelling, appearance of discoloration or bruised skin, and edema of the lymph. Moreover, various medical conditions (e.g., scleroderma; a connective tissue disease) that increases sensitivity of the skin may happen in patients undergoing the radiotherapy [31].

## Curcumin (Cur)

To search for alternative viable options for treatment of BC, many researchers have exploited the biomedical efficacy of naturally originated constituents. Curcumin (Cur) is one of the most-studied natural compounds with a wide range of biomedical applications. Cur is a hydrophobic polyphenol extracted from an herbal dietary spice “Turmeric” which is derived from the rhizome of *Curcuma longa*. Cur has been well-exploited against different types of ailments including the cancer [32–34], wound healing [35, 36], anti-inflammatory [37, 38], antimicrobial [39], antioxidant, antipyretic, bone diseases, and many other illnesses.

In addition to its traditional uses for the management of various health conditions, Cur has shown tremendous anticancer potential against different types of cancer including the BC [40]. Cur is a potent anticancer agent alone or in conjunction with other conventional anticancer therapies such as radiotherapy [41, 42], surgical intervention [43], or chemotherapy [44]. When employed in conjunction with radiotherapy, Cur acts as a potent radiosensitizer for the BC cells/tissues and radioprotector for the normal healthy cells. Though the data is scarce, but many studies have reported that potency and effectiveness of radiotherapy was markedly improved when used in combination with Cur (through oral, subcutaneous, or IV routes) [41, 42]. It has also been proposed that administration of Cur in BC patients prior to the surgical procedure causes shrinkage of the tumor size which results in better therapeutic outcomes [43]. Moreover, substantial data exist in the literature which establishes the synergistic efficacy of Cur and chemotherapy with significant improvement in patient’s quality of life, overall survival rate, low remission, and poor chemoresistance [44]. The anticancer effects of Cur can be attributed to its potent anti-proliferative potential against the BC cells by arresting their cell cycle (G2/M) and induction of apoptosis (p53-dependent) [45, 46]. Downregulation in the expression of EZH2 gene (enhancer of zeste homolog-2) via the mitogen-activated protein kinase (MAPK) pathway also contributes to the anticancer effect of Cur [47, 48]. Cur has also shown an exceptional ability to suppress the proliferation, migration, and invasion in BC cells via the repression of NF-κB [49] and/or downregulation of miRNA-34a that is responsible for epithelial-mesenchymal transition in the tumor microenvironment [50]. Inhibition of Ki-67, proliferating cell nuclear antigen (PCNA), and Bcl-2 as well as upregulation of P-53 mRNA expression and induction of Bax mRNA expression have also been observed in BC cells treatment with Cur [40]. In addition, prevention of angiogenesis (neo-vascularization) due to downregulation of vascular endothelial growth factor (VEGF) has also been evidenced as chemotherapeutic mechanism of Cur against the BC [51, 52].

Despite promising anticancer potential, product development for Cur is hampered due to low aqueous solubility, chemical instability, photo-degradability, low bioavailability [53], rapid metabolism [54] and short-plasma half-life [55], and vulnerability to auto-oxidation [56, 57]. To overcome these challenges, nanotechnology has been deployed for product development of Cur and results have shown revolutionary improvement in the physicochemical properties (e.g., aqueous solubility), chemical stability, and biomedical efficacy of Cur for the management of BC.

## Nanotechnology: Nanoencapsulation of Cur

**Fig. 4:**
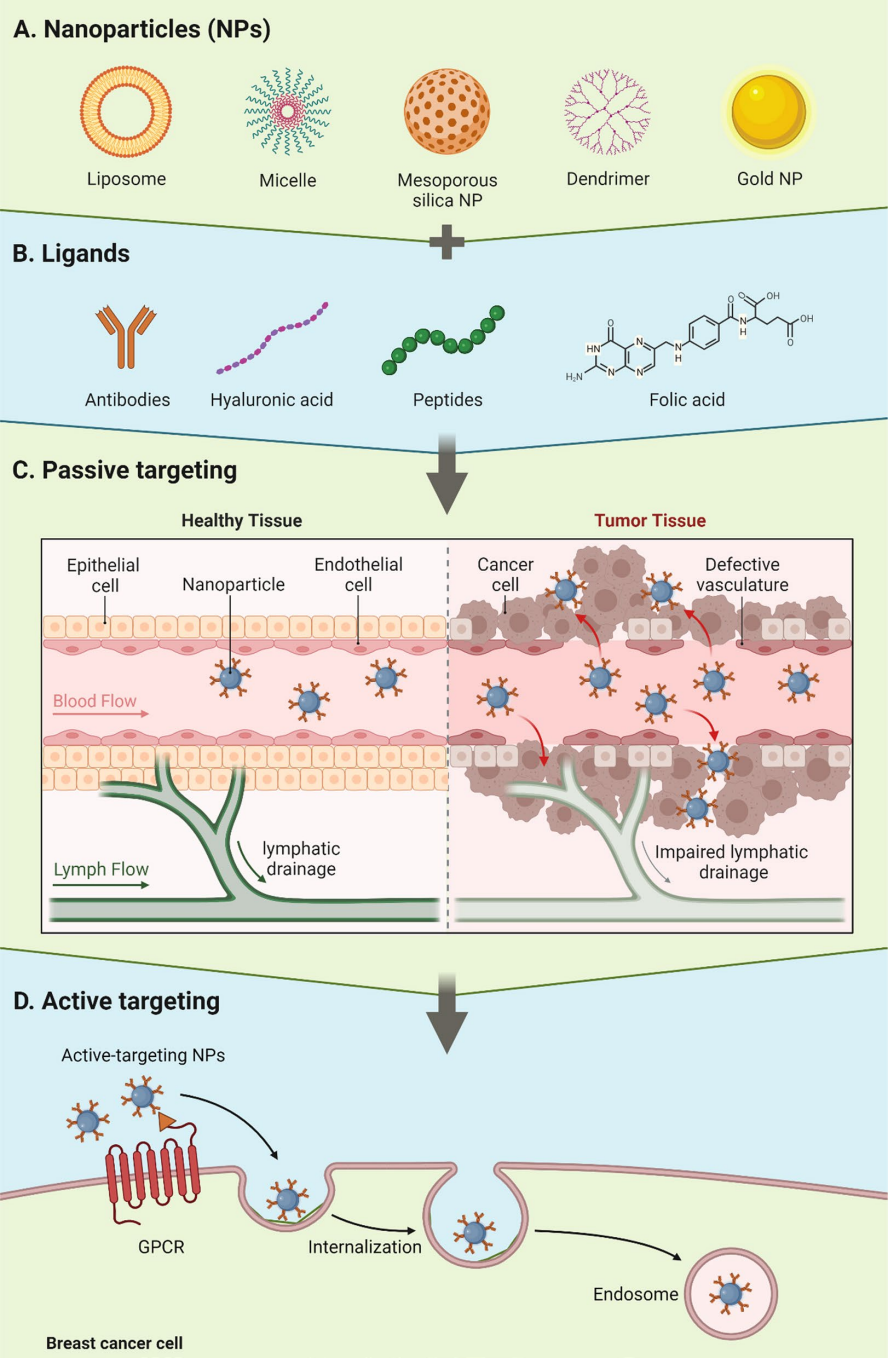
Active and passive targeting approaches for Cur-nanomedicines. Created with BioRender.com

Nanotechnology deals with the synthesis, characterization, and application of nano-scaled materials (1–1000 nm). The deployment of nanotechnology in medicine has shown tremendous potential for early and accurate diagnosis as well as for rational treatment of various diseases including the cancer. The extensive research has been carried out on nanotechnology to improve aqueous solubility, absorption, permeation, bioavailability, and anticancer efficacy of Cur [58–64]. It has been established that a good control over the physicochemical properties (i.e., size, zeta potential, thermodynamics, morphology, and colloidal stability) of Cur-based nanomedicines is mandatory for improving its pharmacokinetic profile and anticancer efficacy [62, 63]. Nevertheless, the nanoencapsulation of Cur has significantly improved its internalization, cell uptake, and anticancer efficacy against BC [64]; however, one of the major limitations of these nanomedicines is lacking of selective targeting which still cause severe side effects. To mitigate this issue, several adaptations have been made in the design of nanomedicines which significantly improve specific targetability of these delivery systems to the tumor microenvironment. Nanomedicines can target the tumor tissues by (a) passive targeting and/or (b) active targeting (Fig. 4).

### Passive targeting

Passive targeting refers to preferential accumulation of Cur-nanomedicines into neoplastic tissues as result of enhanced permeability and retention (EPR) [65]. The passive accumulation of Cur-nanomedicines into tumor microenvironment (TME) can be obtained by optimizing their physicochemical features such as ultra-fine particle size, hydrophilic exterior, good zeta potential, and surface functionalization with various hydrophilic moieties. Another important factor which is responsible for the passive targeting of Cur-nanomedicines into TME is the leaky vasculature of tumor tissues due to irregularly arranged endothelial cells in the newly formed blood vessels because of the abnormal angiogenesis [66, 67]. One of the reasons for the leaky vasculature is an imbalance between the supply and demand of the nutrients to the growing and proliferating BC cells. Cancer cells proliferate in an uncontrolled manner and thus have huge demand of nutrients supply which surpass the ability of our body. This results in formation of immature and leaky vasculature and improperly arranged endothelial cells which ultimately results in enhanced permeation of Cur-nanomedicines into the tumor tissues from the blood circulation. In addition, the cancerous tissues are unable to build mature and proper lymphatic system which results in poor drainage of the permeated nanomaterials and as a result prolongs the retention of Cur-nanomedicines into the cancerous tissues.

### Active targeting

Active targeting refers to the selective delivery of drugs to the specific cells/tissues of the body [68]. To achieve this, various targeting ligands (i.e., peptides, antibodies, folic acid, hyaluronic acid, etc.) can be conjugated on the surface of Cur-nanomedicines which facilitate the recognition of specific substrate receptors (e.g., folate, CD44, transferrin receptors, etc.) that overexpress on the surfaces of tumor cells [68]. The specific interaction of targeting ligand conjugated nanomedicines with receptor-bearing cancer cells results in a selective accumulation of Cur-nanomedicines into the target cancer cells/tissues and as a result off-target accumulation of chemotherapeutic payload is averted (Fig. 4).

## Cur-nanomedicines and adaptable functionalizations

**Fig. 5:**
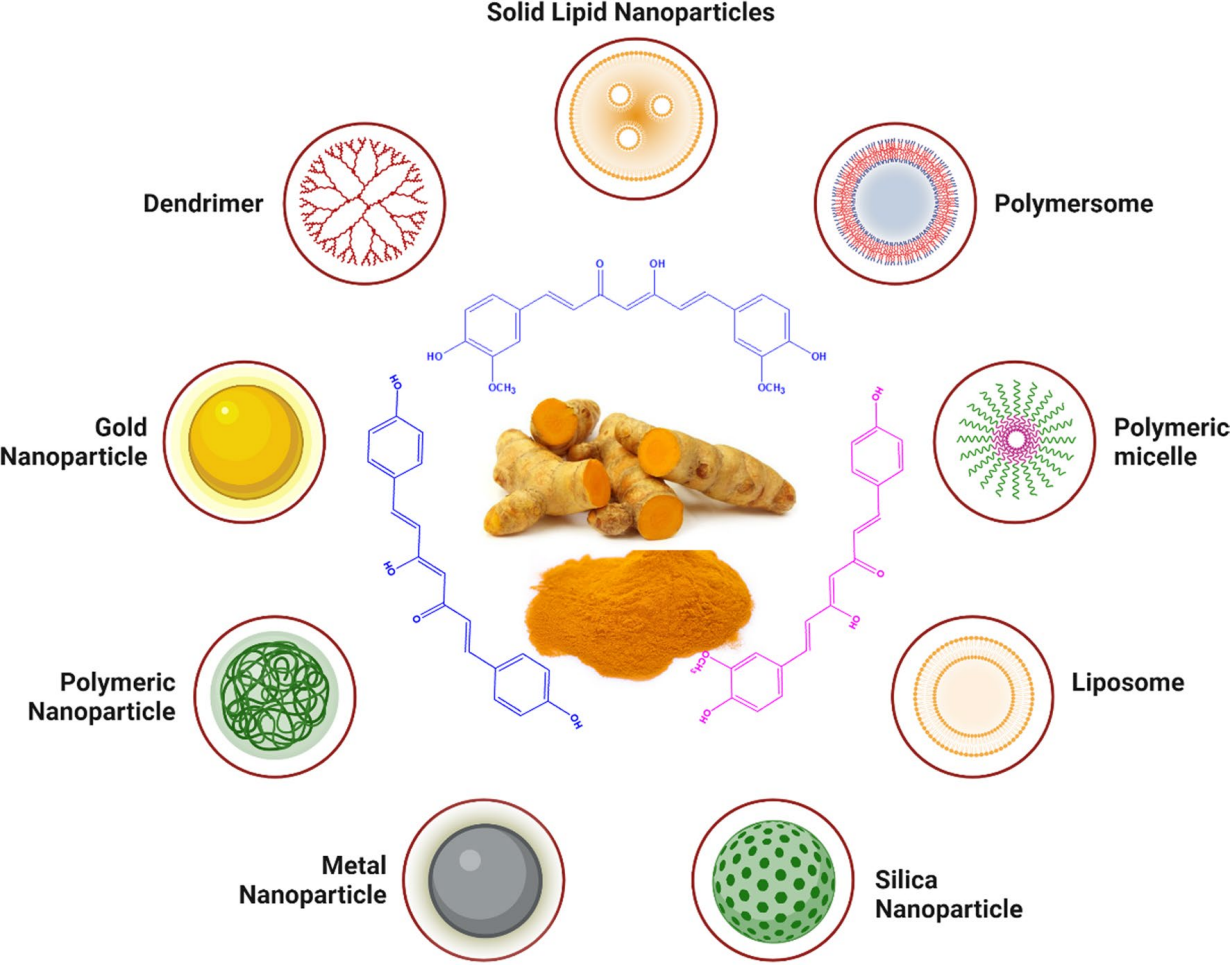
Types of Cur-nanomedicines exploited for the treatment of BC. Created with BioRender.com

In the recent decades, a variety of nanodelivery systems have been engineered for improving the physicochemical properties, pharmacokinetic profile, biodistribution (via passive or active targeting), and anticancer efficacy of Cur against the BC (Fig. 5). Nonetheless, some Cur-nanomedicines have satisfactorily addressed almost all the issues associated with the Cur; however, majority of Cur-nanomedicines are still facing grandeur challenges particularly in in vivo settings and in humans, which restrict their clinical translation. Therefore, in the following sections, we have critically discussed a variety of Cur-nanomedicines as well as diverse adaptations (functionalizations) that have been carried out in the architecture of Cur-nanomedicines to mitigate challenges and to further improve their pharmacokinetic profile and anticancer efficacy against the BC.

### Liposomes

Owing to their structural resemblance (lipid bilayer) with the biological membranes, liposomes (spherical-shaped nanovesicles) have been extensively employed as a drug nanocarrier for a wide variety of drugs [69–71]. Moreover, the high encapsulation efficiency, ability to encapsulate the hydrophilic and hydrophobic drugs simultaneously, biocompatibility, good permeation efficiency, sustained release characteristics, and high flexibility of modulation make liposomes an efficient delivery vehicle. Due to their unique architecture and physicochemical features, liposomes have significantly improved aqueous solubility, absorption, bioavailability, biodistribution, and anticancer efficacy of Cur. However, recent developments including the conjugation of targeting ligand(s) on the exterior surface of the liposomes for active targeting, PEGylation (stealthing) to prolong the plasma half-life, and incorporation of the pH-sensitive linker have profoundly improved the pharmacokinetic profile and anticancer efficacy of Cur [70, 71].

Hasan and colleagues [72] fabricated the Cur-encapsulated liposomes and evaluated for physicochemical properties and cytotoxicity against the BC cells (MCF-7). A dose dependent increase in the cytotoxicity was observed in MCF-7 cells treated with Cur-liposomes compared to the free Cur. The cytocompatability test against the normal breast epithelial cells (MCF-10A) validated that Cur-liposomes showed no signs of cytotoxicity in these non-malignant breast cells which indicated their selectivity towards BC cells. The anticancer efficacy of Cur-loaded liposomes was attributed to upregulated production of reactive oxygen species (ROS) and substantial damage to essential sub-cellular structures (i.e., DNA, RNA, and proteins) of the BC cells [72]. Furthermore, the surface functionalization of Cur-liposomes with salmon's lecithin showed superior anticancer efficacy compared to the Cur-liposomes without surface functionalization as well as the ones functionalized with rapeseed and soya lecithins. These results concluded that functionalization of Cur-liposomes with salmon's lecithin significantly improves their properties and selective targeting of BC cells [72]. Same research group later also reported that physicochemical properties, colloidal stability, oral bioavailability, and anticancer efficacy of Cur against the BC can be further augmented via the functionalization of Cur-liposomes with chitosan (CS) [73, 74]. Likewise, the complexation of Cur with β- or γ-cyclodextrin (CD) with subsequent encapsulation into the liposomes was also found promising strategy for increasing the aqueous solubility and physicochemical stability of the Cur [75, 76].

**Fig. 6:**
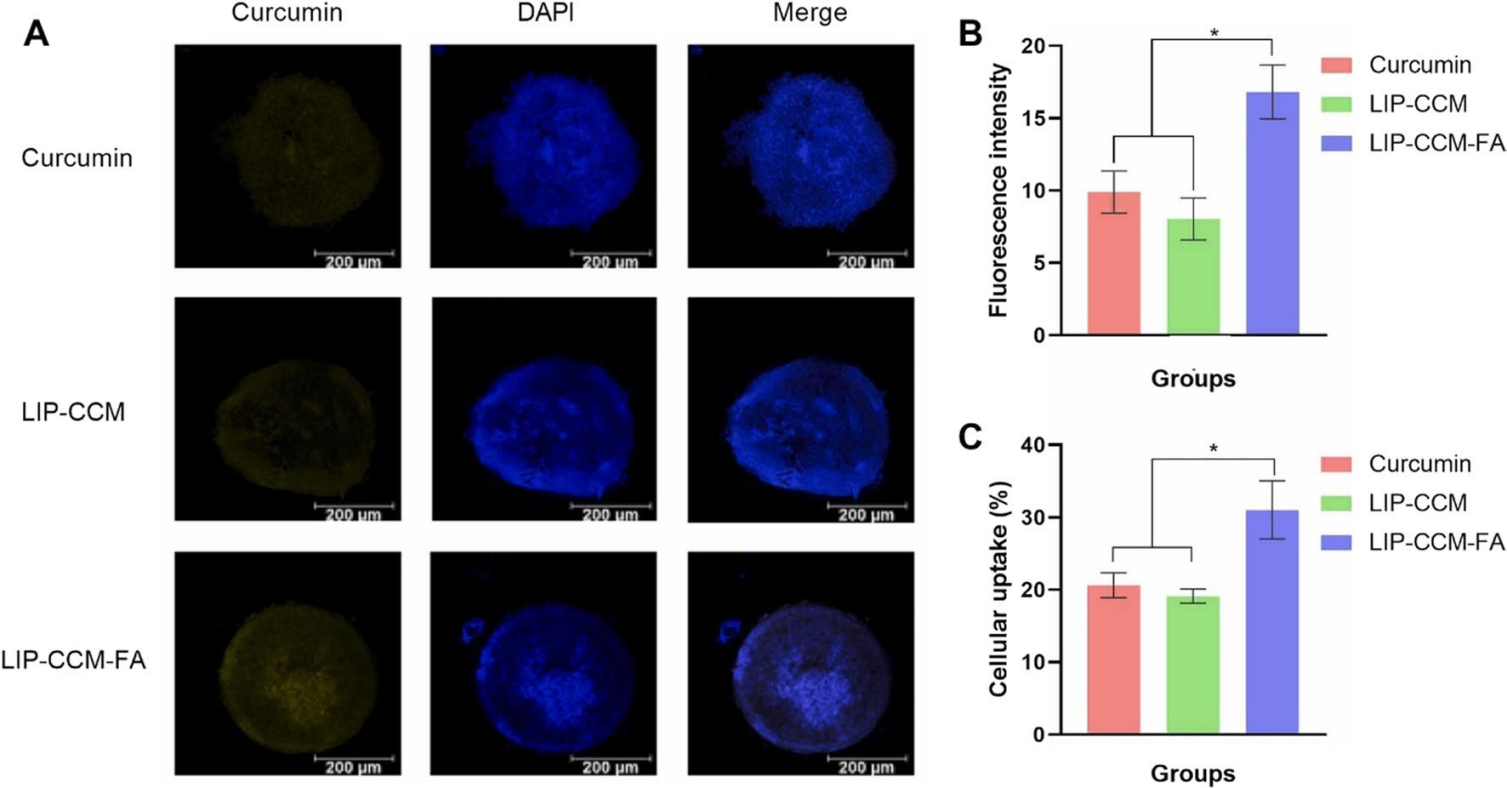
Cell uptake efficiency of FA-functionalized CUR-loaded liposomes (LIP-CCM-FA): **A** confocal images of MCF-7 taken at 24 h post-incubation, **B** fluorescence intensity, and **C** cell uptake efficiency. Adapted from [79]

Active targeting strategy has gained an exceptional recognition in mitigating the off-target effects of the chemotherapeutic agents. Therefore, extensive research has been carried out on functionalization of liposomes with specific ligands such as folic acid (FA) [77]. In this study, the specific cell uptake efficiency of FA-functionalized Cur-liposomes was evaluated using the malignant triple negative BC cells (MDA-MB-231) compared to the non-malignant breast cells (MCF-12A). A strong fluorescence observed in the MDA-MB-231 cells compared to the MCF-12A cells indicated the selective targetability of FA-functionalized liposomes which was attributed to overexpressed FA-receptors on the surface of malignant BC cells [77]. It has also been reported that LD_50_ of Cur-liposomes in MDA-MB-321 was around 19 µM which was significantly lower than LD_50_ observed in MCF-12A. Conclusively, FA-conjugation of Cur-liposomes can be a promising adaptation to improve the specific targetability and anticancer efficacy of Cur-liposomes against the BC [78]. The selective targeting efficiency of FA-functionalized Cur-liposomes has also been investigated by the Luiz and colleagues [79]. The fabricated liposomes exhibited ultra-fine particle size (138 nm), good encapsulation efficiency (⁓73%), and smooth spherical morphology. FA-functionalized Cur-liposomes (LIP-CCM-FA) also displayed a significant improvement in the cytotoxicity, cell uptake efficiency (higher fluorescence), and anticancer efficacy against the BC cells (MCF-7) compared to the unfunctionalized Cur-liposomes and the plain Cur (Fig. 6) [79].

Another biochemical target that has been well-studied by the researchers for selective targeting of BC cells is the human epidermal growth factor receptor-2 (HER-2). Hence, many researchers have designed unique Cur-liposomes for specific targeting of HER-2 receptors overexpressed on the surface of BC cells. Moballegh-Nasery et al. [80] engineered affibody-decorated Cur-loaded liposomes and investigated anticancer efficacy against the BC cells (SKBR3 and MCF7). The fabricated Cur-loaded liposomes (⁓150 nm) displayed a significant improvement in cell uptake efficiency, cytotoxicity (via induction of apoptosis), and anticancer efficacy [80]. Another functionalization for improving the aqueous solubility, oral absorption, and anti-tumor efficacy of Cur against the BC was surface coating of Cur-loaded bilosomes with D-alpha-tocopherol polyethylene glycol succinate (TPGS) (TPGS-Cur-Bil) [81]. The fabricated TPGS-Cur-Bil possessed the nanoscaled size (⁓190 nm), narrow PDI (0.26), good zeta potential (−41 mV), high encapsulation efficiency (93%) and good storage stability. In comparison to the free Cur and unfunctionalized liposomes (Cur-Bil), the TPGS-Cur-Bil displayed a significantly higher cell uptake efficiency and cytotoxicity against the MCF-7-ADR which evident the promising potential of TPGS-functionalization for specific targeting and enhanced anticancer efficacy against the breast cancer [81].

Hybrid nanomaterials which are unique chemical conjugates of different materials have gained special recognition due to their diverse unique properties. Ruttala et al. [82] designed a novel hybrid PEGylated liposome encapsulated with PTX-loaded albumin-NPs and Cur. The purpose of this hybrid nanosystem was simultaneous delivery and sustained release of two chemotherapeutic agents (PTX and Cur) for the synergistic anticancer efficacy. The developed PEGylated hybrid liposomes were characterized and evaluated for cytotoxicity against BC cells (MCF7 and B16F10), cell uptake efficiency, and antineoplastic efficacy. The PEGylated hybrid liposomes exhibited nanoscaled dimension (⁓200 nm), narrow PDI, high encapsulation efficiency (99%), smooth spherical morphology, and good storage stability in the serum at both 4 °C and 25 °C. The Cur-PTX co-encapsulated hybrid liposomes (CL-APN) also displayed a significant increase in the cell uptake efficiency with subsequent reduction of cell migration in both BC cells (MCF7 and B16F10) compared to the plain Cur-liposomes and the pure Cur. These results indicated that multi-functionalization is the recent-most adaptation for improving the anti-proliferative and anti-metastatic efficacy of Cur-liposomes for the treatment of BC [82].

As functionalization of Cur-nanomedicines is relatively a newer adaption, therefore, no patent is granted yet to these nanoformulations. However, a patent was granted to Kurzrock and colleagues on Cur-loaded liposomal formulation developed for the treatment of a variety of cancers including the BC, pancreatic cancer, and melanoma [83]. The encapsulation of Cur into the liposomes resulted in a significant improvement in the aqueous solubility, chemical stability, cytotoxicity, cell uptake, antioxidant potency, and anticancer efficacy against different types of cancer including the BC [83].

### Solid lipid nanoparticles (SLNs)

Due to their ultra-fine particle size, composition, thermodynamic stability, biocompatibility, and flexibility of modulation, the SLNs can significantly improve delivery of anticancer drugs. Nanoencapsulation of drugs into SLNs provides good protection against the chemical degradation and hence improves the storage stability of Cur [84]. SLNs also prolong the blood circulation time which result in improvement of pharmacokinetic profile, biodistribution to cancer tissues (via EPR effect), and improvement of therapeutic efficacy [85, 86]. Rahman and colleagues [84] developed the SLNs encapsulated with niclosamide (an oral anthelminthic drug) for improving its aqueous solubility, absorption, and oral bioavailability. Results showed a sustained release of encapsulated drug (⁓93% release occurs within 12 h) and improved oral bioavailability (2.2-fold higher than the pure drug) [84]. Similarly, melphalan-loaded SLNs were developed for systemic delivery [85]. Results showed a promising improvement in the aqueous solubility, chemical stability, biocompatibility, and a sustained release profile. The drug loaded SLNs also displayed an extended half-life, lower plasma clearance, and higher drug retention into the target tissues [85]. Keeping in view of their great stability, affordability, scalability, and biopharmaceutical feasibility, many researchers have employed SLNs for targeted delivery of CUR for the treatment of BC.

The physicochemical properties, stability, and transcellular permeation of SLNs is greatly influenced by the type(s) and nature of the lipid ingredient(s) used to formulate the SLNs. Therefore, Wang and coworkers [86] developed Cur-encapsulated SLNs by using two lipids (stearic acid and lecithin) and evaluated their efficacy for treatment of BC (SKBR3 cells). The developed Cur-SLNs exhibited the spherical morphology with 30–50 nm size and −25.3 mV zeta potential which indicates good thermodynamic stability of these NPs. The cytotoxicity data showed an IC_50_ of 18.78 µM of Cur-SLNs compared to the IC_50_ of 28.42 µM of free CUR after 48 h incubation with SKBR3 cells which clearly reflects an enhanced anti-proliferative efficacy of Cur-loaded SLNs. The cell uptake study displayed a relatively higher internalization of Cur-SLNs (higher fluorescence intensity) into the SKBR3 cells compared to the free Cur. The higher cell uptake of Cur-SLNs was expected to be one of the reasons for superior anticancer efficacy of Cur-SLNs compared to the free Cur. The anti-proliferative effect of Cur-SLNs was attributed to arresting of cell cycle at G1/S phase [86] and inducing the apoptosis via generating the ROS which upregulates the apoptosis by damaging the mitochondrial membrane via depolarization [85–87]. Likewise, Bhatt et al. [88] fabricated Cur-SLNs using a single lipid (glyceryl monostearate) in the presence of Poloxamer 188 as a stabilizer (surfactant). They optimized the Cur-SLNs via the quality-by-design (QoD) approach and tested the anticancer efficacy of optimized Cur-SLNs against the BC cells. The fabricated Cur-SLNs exhibited nanoscaled particle size (⁓230 nm), narrow PDI, and good loading efficiency. The comparative analysis revealed that Cur-SLNs displayed a superior cell uptake efficiency, downregulated viability, and significantly higher apoptosis in BC cells (MDA-MB-231) compared to the free Cur [88]. The Cur-SLNs have also been synthesized using the cholesterol as the lipid ingredient in the presence of Poloxamer 188 [89]. The optimized Cur-SLNs exhibited ultra-fine particle size (⁓170 nm), narrow PDI, and high encapsulation efficiency. The Cur-SLNs displayed a significantly higher anticancer potential in terms of downregulation of cell viability, higher cell uptake, and enhanced apoptosis in the BC cells (MDA-MB-231) compared to the free Cur [88]. Similar findings have also been reported by other studies [90–92].

One of the prime challenges in the treatment of BC is its aggressive nature and greater metastatic potential. The radiotherapy is one the viable options for the management of local, inoperable, incompletely resected, and recurrent BC. Therefore, Minafra et al. [93] proposed a synergistic therapy with Cur-SLNs (as a radiosensitizer and synergistic molecule) and radiotherapy (2–9 Gy) for the superior therapeutic outcomes against the BC. The therapeutic potential of adjuvant therapy (Cur-SLNs + radiotherapy) was tested against the tumorigenic BC cells (MCF7 and MDA-MB-231) and the non-tumorigenic BC cells (MCF-10A). Interestingly, adjuvant therapy (Cur-SLNs + radiotherapy) displayed a significantly higher cytotoxicity (lower IC_50_) compared to the radiotherapy alone or in combination with free Cur. These results were attributed to Cur-SLNs which enhanced the radio-sensitization of BC cells against the radiation therapy as well as enhanced antioxidant potency [93].

Active targeting approach has also been adapted in the design of SLNs for selective targeting of BC cells/tissues [94–96]. Like other phenotypic markers, transferrin receptors are also overexpressed on the surface of different types of cancer cell including the BC cells. Therefore, Mulik et al. [97] developed transferrin conjugated SLNs for targeted delivery of Cur-SLNs into BC cells. The prepared transferrin-conjugated Cur-SLNs exhibited an ultra-small particle size, good encapsulation efficiency, sustained release profile and a significant improvement in cell uptake into MCF7 cells compared to the unfunctionalized Cur-SLNs and the free Cur. These results concluded that conjugation of Cur-SLNs with transferrin could be a promising approach to maximize the selective targeting into BC cells with ultimately enhanced anticancer efficacy against the BC [97]. On the other hand, Pawar et al. [98] developed SLNs for the co-delivery of Cur and docetaxel (DTX) and functionalized with FA. Additionally, they coated the exterior surface of FA-Cur-SLNs with polyethylene glycol (PEG) to confer them immune-compatible and biocompatible. The PEGylation was aimed to avert the recognition of Cur-SLNs by the RES for prolonging the plasma circulation time. The fabricated FA-conjugated Cur/DTX-SLNs were investigated for cytotoxicity against the BC cells (MDA-MB-231 cells) and for cell uptake efficiency (MCF-7). The optimized FA-Cur/DTX-SLNs exhibited an ultra-fine particle size (180–250 nm), good zeta potential (−27.5 mV), high encapsulation efficiency (⁓72%), smooth spherical morphology, and biphasic release profile. The FA-conjugated SLNs also displayed significantly higher cell uptake efficiency and lower cell viability compared to the unfunctionalized Cur-SLNs and the free Cur. Moreover, better pharmacokinetic and biodistribution profile was evidenced in case of functionalized Cur-SLNs compared to the unfunctionalized Cur-SLNs and the free Cur [98]. These results were also in agreement with another study [99]. These findings indicate that surface functionalization of Cur-SLNs via PEGylation or conjugation of targeting ligand(s) enhance their cell uptake efficiency, cytotoxicity, pharmacokinetic profile, biodistribution, and anticancer efficacy against the BC.

### Albumin-NPs

By considering their biocompatibility and non-antigenicity, the albumin NPs have been broadly used for treatment of different diseases including the BC. Like other nanocarriers, the physicochemical features (i.e., particle size, surface chemistry, entrapment capacity, morphology, and colloidal stability) of the albumin-NPs play an imperative role in their transmembrane permeability, biodistribution, and specific targeting to the TME. Keeping in view of the great potential of albumin NPs, Jithan et al. [100] developed albumin-NPs for improving the plasma circulation time, preferential distribution to TME, and the anticancer efficacy of Cur. The prepared Cur-loaded albumin-NPs exhibited a nano-scaled size (< 250 nm), good encapsulation efficiency (75%-85%), and highly sustained release kinetics (90% Cur was released in one month) [100]. The Cur-loaded albumin-NPs also displayed significantly higher cytotoxicity against the MDA-MB-231 cells compared to the free Cur. The superior pharmacokinetic profile, biodistribution to tumor tissues, and anticancer efficacy of Cur-loaded albumin-NPs has also been validated in vivo in comparison with free Cur [100].

To further improve their selective targeting and anticancer efficacy, a variety of functionalization methods have been adapted in the design of Cur-loaded albumin-NPs. Hasanpoor et al. [101] engineered Cur-loaded albumin-NPs (human serum albumin, HSA) and decorated their exterior surface with PDL-1 (programmed death ligand-1) to confer them with selective targeting feature. Like other phenotypic markers, BC cells also exhibit over-expression of PD-L1 which is largely responsible for evasion of immune response and thus contribute to chemoresistance in BC. The developed PDL-1 functionalized Cur-loaded albumin-NPs (P-HAS/Cur-NPs) were evaluated for cell uptake efficiency and dose dependent (12.5, 25, 35, and 50 μM) cytotoxicity against the BC cells (MCF-7 and MDA-MB-231) at different time periods (24, 48, and 72 h). The fabricated P-HAS/Cur-NPs exhibited 200–250 nm particle size with smooth spherical morphology. The cell cytotoxicity study depicted a time- and dose-dependent cytotoxicity in both the BC cells treated with P-HAS/Cur-NPs compared to the unfunctionalized albumin-NPs (HAS/Cur-NPs) and the free Cur. Interestingly, the IC_50_ (61 μM) of P-HAS/Cur-NPs PD against the MDA-MB-231 cells at 72 h was fourfold less than the IC_50_ (234 μM) obtained with free Cur which indicates a powerful cytotoxic response of peptide-conjugated Cur-loaded albumin-NPs against the BC cells [101]. The P-HAS/Cur-NPs also exhibited higher cytotoxicity against the MCF7 cells compared to the unfunctionalized HAS/Cur-NPs and the free Cur. As expected, the cell uptake study (florescent microscopy and flow cytometry) also validated superior ability of PDL-1 conjugated albumin-NPs (P-HAS/Cur-NPs) to internalize into BC cells (higher fluorescence) compared to the unfunctionalized NPs (HAS/Cur-NPs) and free Cur. These results evidenced that PDL-1 decoration on the surface of Cur-loaded albumin-NPs can be a promising adaption to maximize the selective targetability and anticancer efficacy against the BC [101].

On the other hand, Thadakapally and research group [102] proposed the PEGylation of Cur-loaded albumin-NPs to prolong their plasma circulation time, enhance the passive accumulation into BC cells and to reduce the hepatic clearance. The anticancer efficacy of prepared NPs (PEG-Cur-albumin-NPs) was tested against the BC cells (MD-MB-231). A biphasic release pattern was exhibited by the developed PEG-Cur-albumin-NPs with initial burst release followed by the sustained release over 35 days. The cell uptake study indicated a significantly higher uptake efficiency of PEGylated NPs into MD-MB-231 cells compared to the unPEGylated albumin-Cur-NPs and the free Cur [102]. In comparison with unfunctionalized albumin-Cur-NPs and the free Cur, lower uptake of PEG-albumin-Cur-NPs by the liver and kupffer cells indicated a marked stealthing effect of PEGylation. These findings indicated that PEGylation of Cur-loaded albumin-NPs could be a promising adaption to improve the plasma circulation time, preferential accumulation into BC cells (via EPR effect) and enhanced anticancer efficacy against the BC [102].

Recently, a newest design called “multifunctionalized NPs” have been developed by the Hiremath et al. [103] for the co-delivery of Cur and 5-flurouracil (5-FU) and evaluated against the BC cells (MCF7). Briefly, the Cur/5-FU co-loaded iron-oxide NPs were synthesized and decorated with albumin (to confer them with stealthing effect and to prolong the plasma half-life) and citrate (to render them a selective targeting feature for receptor-mediated endocytosis into BC cells). Furthermore, FA was conjugated to albumin to maximize the selective targetability to BC cells/tissues. The fabricated multifunctionalized NPs exhibited ultrafine particle size (10–15 nm), good anionic zeta potential (−49 mV), good colloidal stability, and a sustained release profile for both drugs (Cur and 5-FU). The cell uptake study indicated a superior internalization of multifunctionalized NPs (FA-albumin-Cur/5-FU-NPs) into the BC cells compared to the unfunctionalized NPs (Cur/5-FU-NPs) and the free Cur. The FA-albumin-Cur/5-FU-NPs also produced highest cytotoxicity in the BC cells compared to the control groups. These results indicated that multifunctionalization could be a successful evolving strategy to maximize the plasma circulation time, cell uptake, biodistribution to TME, specific targetability, and anticancer efficacy against the BC [103].

### Polymeric nanoparticles (NPs)

Polymeric NPs are among the most extensively studied nanodelivery systems due to their intrinsic features including the particle size, morphology, entrapment efficiency, biodegradability, biocompatibility, colloidal stability, and good flexibility of functionalization [104–110]. Depending upon their architecture, polymeric NPs can be classified into (a) nanosphere and (b) nanocapsules [111]. The nanospheres are polymeric NPs in which drug(s) are encapsulated within the polymeric solid matrix or adsorb on the surfaces; however, in the nanocapsules drug(s) are mainly encapsulated within the inner core which is surround by a solid matrix of polymer. The properties of polymeric NPs largely depend upon the type of polymer used to construct them [111–113]. A wide variety of polymers such as natural, synthetic, and semi-synthetic polymers have been employed to fabricate the polymeric NPs [112–115].

Having considered their intrinsic physicochemical features and diversity of functionalization, Pawar et al. [116] developed PLGA-NPs for co-delivery of Cur and DTX. The developed Cur/DTX-PLGA-NPs were investigated for cytotoxicity, cell uptake, and pharmacokinetic profile (in male Wistar rats). The Cur/DTX-PLGA-NPs exhibited ultrafine particle size (219 nm), narrow PDI, anionic zeta potential (−14 mV), good encapsulation efficiency (⁓67%), and biphasic release kinetics. The cell uptake experiment indicated a significantly higher uptake of Cur/DTX-PLGA-NPs into BC cells (MCF7 cells) compared to the free Cur. The pharmacokinetic study revealed that Cur/DTX-PLGA-NPs displayed fivefold higher AUC in comparison with free drug. These results concluded that co-encapsulation of Cur and DTX into PLGA-NPs could improve their pharmacokinetic profile and anticancer efficacy against the BC [116].

For maximizing the cell uptake efficiency of PLGA-NPs into BC cells (MCF7), Sampath et al. [117] developed PLGA-NPs and functionalized them with a variety of surface functionalizing agents such as PEG, TPGS, chitosan and dextran. The functionalized Cur-PLGA-NPs exhibited ultra-fine particle size (< 250 nm), good loading capacity (> 90%), smooth spherical morphology, and good storage stability. The comparative analysis between different nanoformulations indicated that Cur-PLGA-NPs functionalized with TPGS exhibited highest cytotoxicity via the ROS-induced apoptosis in BC cells. Similarly, the cell uptake experiment also demonstrated highest internalization of TPGS-Cur-PLGA-NPs into MCF-7 cells compared to the Cur-PLGA-NPs functionalized with other moieties (PEG, chitosan, and dextran) and the free Cur [117].

One of the major obstacles for PLGA-NPs as a drug delivery device is the recognition by the RES [118]. Upon recognizing them as foreign materials, RES engulf the PLGA-NPs with subsequent metabolism and excretion from the body via the lymphatic drainage. To avoid RES-mediated recognition and clearance of Cur-PLGA-NPs, various surface functionalization strategies have been adapted. One of these approaches which gained exceptional recognition is the formation of hydrophilic layer around the exterior surface of polymeric NPs using a hydrophilic moiety (e.g., PEG) [119, 120]. This technique has been widely employed for improving the plasma circulation time, pharmacokinetic profile, biodistribution, cell uptake efficiency (EPR effect) and anticancer efficacy of Cur-PLGA-NPs against the BC [117, 121]. The PEG-Cur-PLGA-NPs upregulate ROS-induced apoptosis, alleviate angiogenesis (via inhibition of VEGF expression), reduced proliferative property (cyclin-D1) and great anti-metastatic potential (via declining MMP-9 expression). A strong anti-proliferative efficacy of PEG-Cur-PLGA-NPs was attributed to arresting of cell cycle at G2/M phase in MCF7 cells. The anticancer efficacy of PEG-Cur-PLGA-NPs was also validated in tumor bearing animals which demonstrated an improved bioavailability, an extended plasma half-life, high biodistribution into tumor tissues, and superior anticancer efficacy [121, 122].

Another method of functionalization of Cur-PLGA-NPs was proposed by Palange and co-researchers [123] by coating the surface of NPs with lipids to confer them hydrophobicity for better permeability and uptake into MDA-MB-231 cells. One of the promising causes of high fatality rate in BC patients is high metastatic rate and invasion of BC cells into other organs. BC cells travel from primary tumor sites to other body regions through vascular or lymphatic systems [124]. These circulating tumor cells (CTCs) are capable of translocating from the primary sites (BC tissues) to other body organs and can stay in blood for prolonged period and may affect other normal tissues in a way similar as that leukocyte recruitment [125]. This also results in initiation of inflammation at the site where metastasis occurs [126, 127]. Though, Cur exhibits a potent anti-inflammatory, anti-proliferative, and CTCs migration inhibition efficacy [128, 129]; however, poor bioavailability, low water solubility, and minimal adsorption of Cur [130–132] reduce its anticancer viability. To avoid these problems, Palange and coworkers developed a long-circulating lipid-coated Cur-PLGA-NPs for efficient targeting to CTCs (MDA-MB-231) and inflamed endothelial cells (HUVECs) (TNF-α stimulated human umbilical vein endothelial cells) [123]. The developed NPs consisted of a polymeric core (made up of PLGA) containing Cur and an outer covering containing a mixture of lipids and PEG to stabilize the Cur-PLGA-NPs as well as to extend the plasma half-life. The lipid-coated Cur-PLGA-NPs showed a dose- and time-dependent cytotoxicity in HUVECs and MDA-MB-231 cells which was significantly higher (lower IC_50_) than the free Cur. Moreover, the adhesion of CTCs to endothelial cells (a hallmark of cancer metastasis) was significantly decreased after the treatment with lipid-Cur-PLGA-NPs compared to the unfunctionalized Cur-PLGA-NPs and the free Cur. These findings demonstrate that lipid coating could be a promising adaption in the design of polymeric NPs for improving their penetrability and anticancer efficacy against the BC [123].

**Fig. 7:**
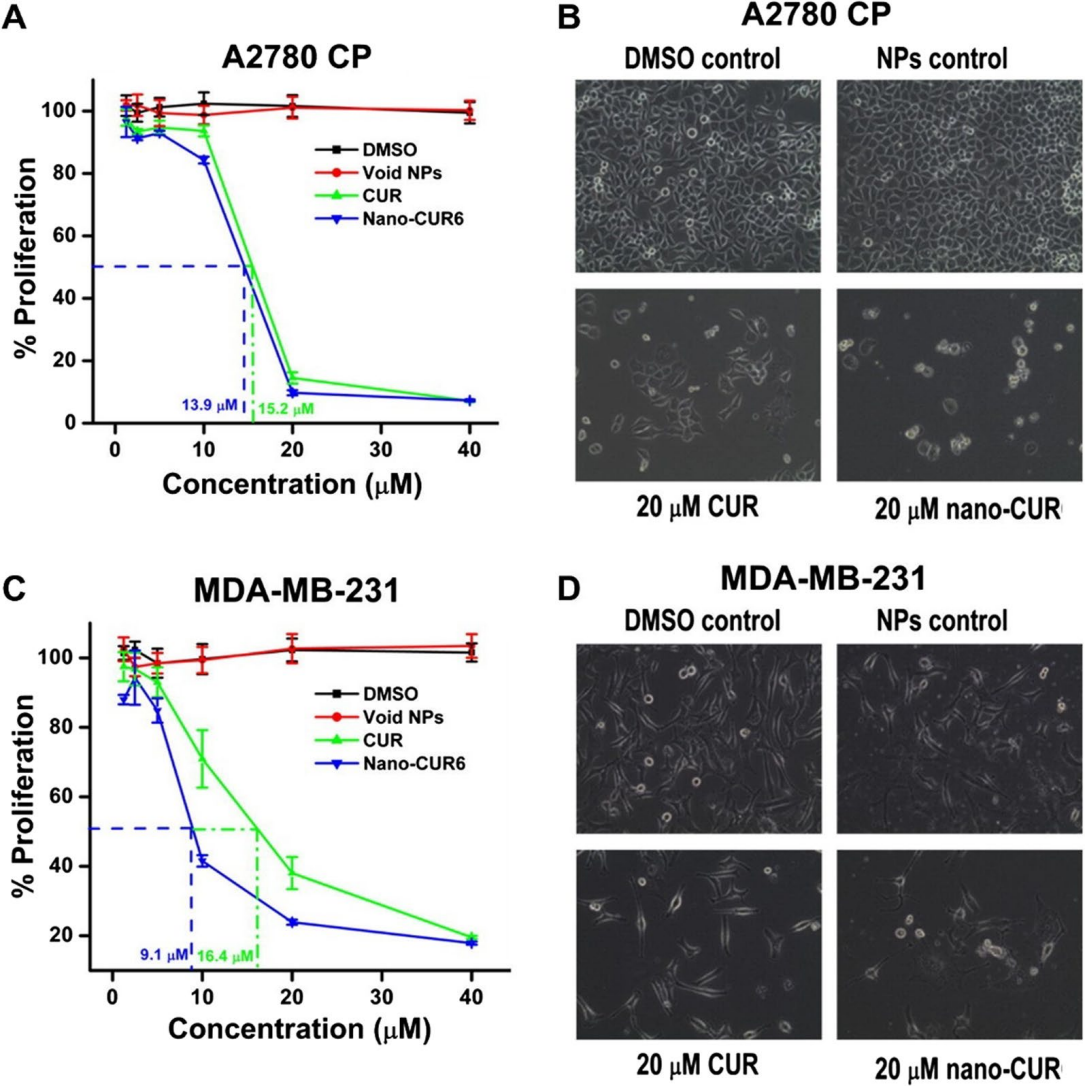
Superior anti-proliferative efficacy of T-Cur-PLGA-NPs (nano-CUR) in ovarian cells (A2780CP) (**A**&**B**) and BC cells (MDA-MB-231) (**C**&**D**) compared to free Cur (20 µM). Adapted from [134]

For selective targeting and receptor-mediator endocytosis, Zheng and co-workers [133] functionalized the exterior surface of Cur-PLGA-NPs with transferrin to recognize and target the transferrin receptors overexpressed on the surface of BC cells. The transferrin-conjugated Cur-PLGA-NPs (T-Cur-PLGA-NPs) showed a significantly higher cell uptake into BC cells (MDA-MB-231) compared to unfunctionalized NPs (Cur-PLGA-NPs) which was attributed to transferrin receptors-mediated endocytosis. The superior uptake of T-Cur-PLGA-NPs was responsible for higher cytotoxicity into BC cells compared to the Cur-PLGA-NPs and the free Cur [133]. Likewise, Yallapu et al. [134] prepared the Cur-PLGA-NPs and tagged with transferrin or anti-TAG-72 monoclonal antibody (Mab) for selective targeting of BC cells. The optimized T-Cur-PLGA-NPs exhibited ultrafine particle size (< 100 nm), narrow PDI, smooth spherical morphology, good encapsulation efficiency, and sustained release profile (up to 25 days). The cell uptake study depicted that T-Cur-PLGA-NPs displayed a superior cell uptake efficiency and anti-proliferative efficacy and lower colony formation in BC cells (Fig. 7) compared to the Cur-PLGA-NPs and free CUR. The superior anti-proliferative efficacy of T-Cur-PLGA-NPs was attributed to enhanced ROS-mediated apoptosis in the BC cells [134]. These results were later confirmed by another research group [135].

Like other biochemical targets, annexin A2 (AnxA2) is also well-studied biochemical target that overexpressed on the surface of BC cells and its expression is very high in aggressive BC cells having great metastatic potential. Ranjan and colleagues were granted a US-patent on the surface functionalization of Cur-loaded PLGA-NPs with AnxA2 antibody. The in vitro testing of AnxA2-Cur-PLGA-NPs against the BC cells (MDA-MB-231) showed a significant increase in the cell uptake efficiency, reduction in cell proliferation, migration, and invasion, metastasis, and growth [136]. The prepared Cur-PLGA-NPs were optimized using the central composite design (Design Expert^®^) for particle size (⁓145 nm), encapsulation efficiency (⁓90%), release profile (⁓60% drug release in 24 h), percent yield (⁓90%), morphology (smooth spherical), and storage condition (stable at 4 °C). These results concluded that AnxA2-functionalization could be a promising adaptation in the design of polymeric NPs for selective targeting of BC cells/tissues [136]. These results were later confirmed by Mukerjee et al. [137].

Another innovative design of Cur-nanomedicines (hybrid Nanocurcumin) which was granted US-patent in 2015 was proposed by Ranjan and colleagues in which they fabricated Cur-loaded PLGA-NPs and encased them into lipid-envelope [138]. The hybrid Nanocurcumin (liposoma-PLGA-curcumin) containing the Cur within the polymeric shell was characterized for particle size, encapsulation efficiency, morphology, release kinetics, cell uptake efficiency, and anticancer efficacy against different cancer types including the BC. The hybrid Nanocurcumin showed significant improvement in bioavailability, cell uptake efficiency, cytotoxicity (lower IC_50_), and potent anticancer efficacy against the BC cells compared to the plain Cur-loaded liposomes and free Cur. Another interesting fact about the hybrid Nanocurcumin was its superior safety profile against the QT-prolongation which is one of the most promising side effects caused by the Cur and other conventional Cur-nanosystems [138].

### Polymeric micelles

**Fig. 8:**
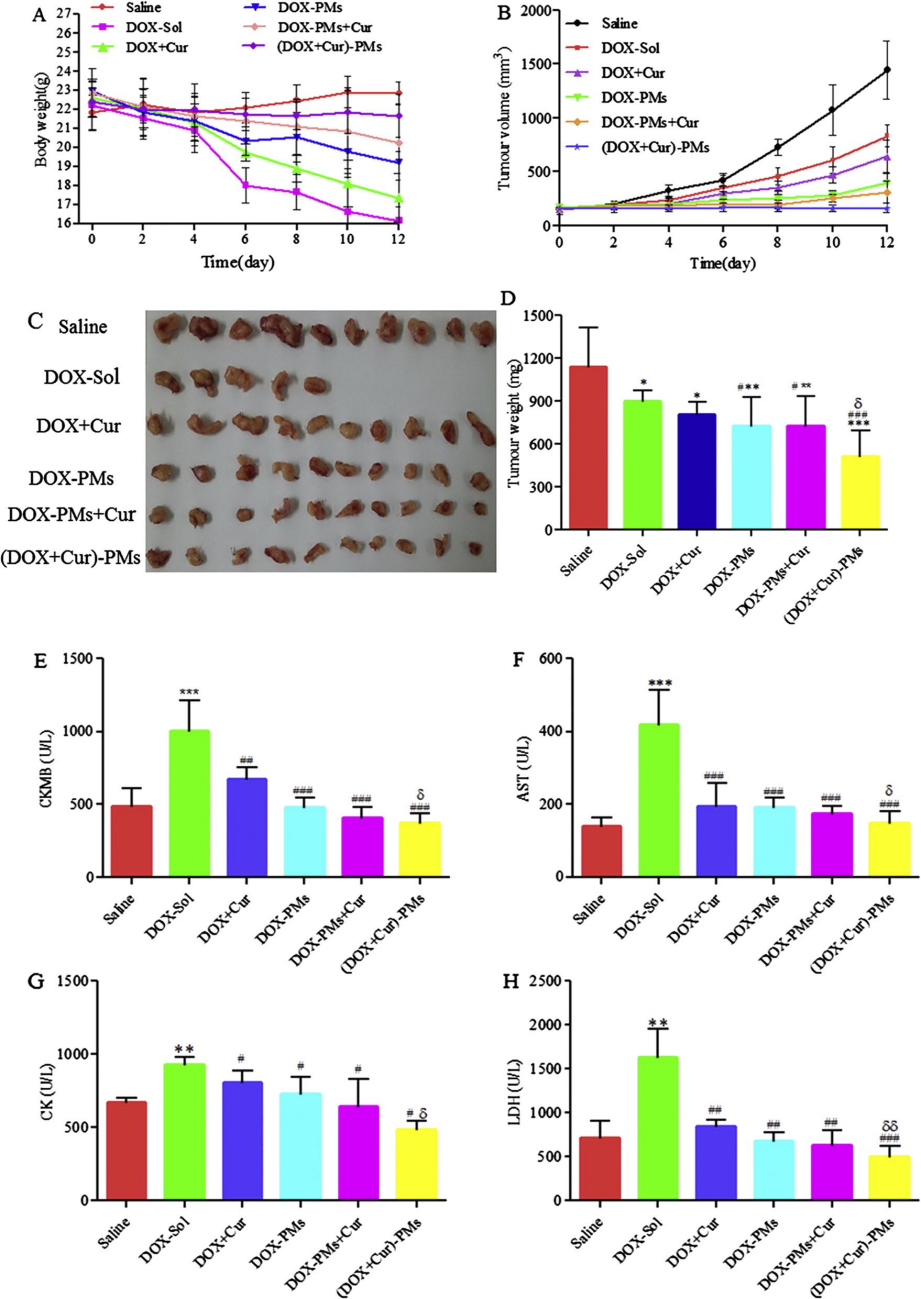
Anticancer efficacy of DOX/CUR co-loaded PMs in 4T1 xenograft mouse model. **A**) relative body weight, **B**) tumor volume, **C**) representative photographs and **D**) average weight of excised tumor harvested from sacrificed mice at the end of the experiment, **E**) serum levels of creatine kinase MB (CKMB), **F**) aspartate transaminase (AST), **G **)creatine kinase (CK), and **H**) lactate dehydrogenase (LDH) after intravenous administration of tested formulations in 4T1-bearing mice. Adapted from [142]

Polymeric micelles (PMs) have been extensively deployed as targeted drug delivery devices for a wide variety of therapeutics particularly for poorly water-soluble drugs [139]. Multi-drug resistance (MDR) is one of the potential hurdles for effective treatment of various types of cancers including the BC [140, 141]; however, to overcome this obstacle, a combination therapy has been adapted for multi-target action. Ma et al. [142] developed hyaluronic acid-vitamin E succinate copolymer-based PMs for simultaneous delivery of Cur and Dox. The developed Dox-Cur-PMs exhibited nanoscaled dimension (⁓223 nm), anionic surface charge (-10 mV), smooth spherical morphology, and high encapsulation efficiency (Cur ⁓72% and Dox ⁓95%). The release study demonstrated that both encapsulated drugs exhibited more prominent and rapid release in an acidic environment (pH 4.5–5.5) compared to the physiologic pH (pH 7.0) which indicate their potential for tumor-specific delivery. On the other hand, in vivo study showed that 4T1 cells bearing mice treated with Dox-Cur-PMs demonstrated greater suppression in the tumor volume compared to other treatment groups. The tumor bearing animals treated with Dox-Cur-PMs displayed a significant suppression of tumor growth rate (⁓55%) compared to the free drugs (Dox + Cur) (⁓28%). The compatibility evaluation of Dox-Cur-PMs evidenced no signs of cardiotoxicity and hepatotoxicity in contrast to the free Dox which induced noticeable cardiotoxicity and hepatotoxicity in the test animals (Fig. 8). These results demonstrate that PMs are promising nanocarriers for co-encapsulation of two chemotherapeutics to improve the anticancer efficacy and reduce the cardiotoxicity and hepatotoxicity associated with Dox [142].

Like other delivery vehicles, ample opportunities for diverse functionalizations are implantable in the design of PMs. Hosseini et al. [143] developed the Cur-loaded PMs for the treatment of BC. To optimize the selective targeting into BC cells, the developed Cur-PMs were functionalized with anti-cyclin-D1 antibody. The cyclin-D1 is a mutagenic gene overexpressed on the surface of BC cells and is responsible for initiation of G1 phase in the cell cycle to promote proliferation of BC cells [144–146]. In addition, cyclin-D1 promotes cell migration, metastasis and increase tumor invasion [144, 145]. The developed anti-cyclin-D1-Cur-PMs (SinaCurcumin^®^) were evaluated for the physicochemical properties, biopharmaceutical properties and therapeutic efficacy against the BC cells (MCF-7) [143]. Results depicted that nanoencapsulation of Cur into PMs resulted in a significant increase in its aqueous solubility and oral bioavailability. The cell cytotoxicity study revealed a pronounced suppression (⁓84%) in proliferation of BC cells treated with SinaCurcumin^®^ compared to the different chemotherapeutic agents such as 5-FU (⁓75%), adriamycin (⁓70%), and cyclophosphamide (⁓63%) [143]. These results suggested that SinaCurcumin® is a more potent anticancer agent compared to the most used anticancer agents such as cyclophosphamide, adriamycin and 5-fluorouracil. It was also established that cyclin-D1 expression was significantly declined in MCF-7 cells treated with SinaCurcumin^®^, demonstrating the downregulation of cyclin-D1 expression on the BC cells. These results concluded that conjugation of anti-cyclin-D1 on the surface of Cur-PMs could be a promising adaption in the design of PMs for the treatment of BC [143].

**Fig. 9:**
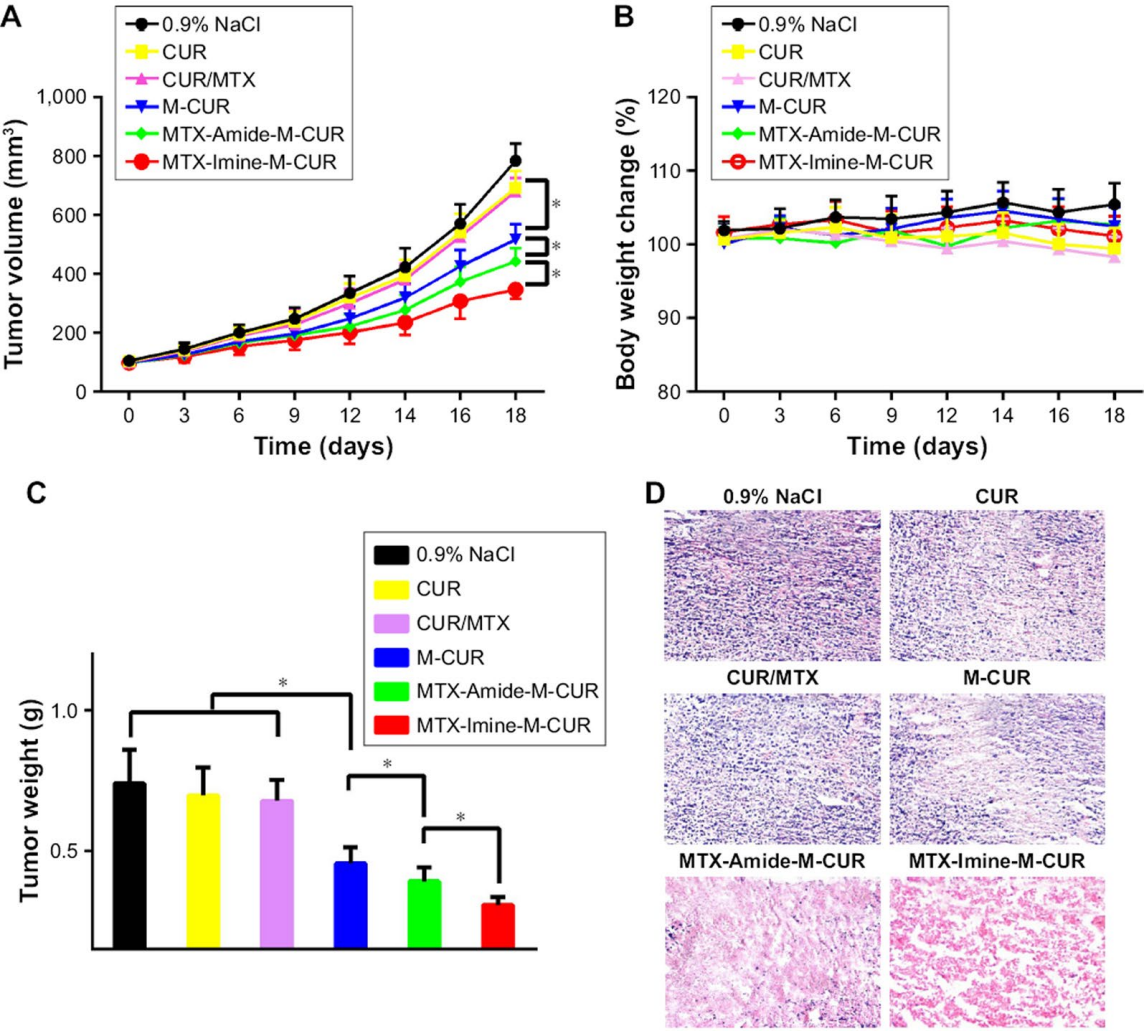
Anticancer efficacy of MTX-imine-M-Cur in HeLa tumor-bearing nude mice after intravenous injection compared to 0.9% NaCl, free Cur, Cur/MTX, M-Cur, and MTX-amide-M-Cur at an equivalent dose of Cur (8 mg/kg). **A**) tumor volume, **B**) body weight, **C**) average weight of excised tumor, and **D**) H&E histologic images of tumors resected from experimental animals. Adapted from [147]

Another innovative method of functionalization of Cur-PMs was reported by the Xie et al. [147] in which dual role of methotrexate (MTX) was proposed. Besides demonstrating a fair anticancer efficacy, MTX possess moderate targeting efficiency (as targeting ligand) for tumor cells having overexpressed FA-receptors due to its structural similarity with FA. In this study, MTX was tagged on the surface of Cur-PM via imine linkage (acid-sensitive linkage which hydrolyze in an acidic environment of TME). The developed Cur-loaded MTX-imine-PMs (MTX-imine-M-Cur) were evaluated for cytotoxicity, cell uptake efficiency, and in vivo anticancer efficacy against the HeLa tumor-bearing BALB/c nude mice. The fabricated MTX-imine-M-Cur exhibited ultrafine particle size (20–30 nm), anionic surface charge, good encapsulation efficiency, smooth spherical morphology, and good colloidal stability. As expected, Cur and MTX release was pronounced at acidic pH (5.0) compared to physiological pH (7.4) which evidence the site-specific release behavior of MTX-imine-M-Cur. The cell culture study revealed a significantly higher uptake efficiency and greater cytotoxicity in BC cells (MCF-7) compared to the control groups. The tumor-bearing animals treated with an intravenous injection of MTX-imine-M-Cur (equivalent to 8 mg/kg) displayed a significantly higher anticancer efficacy in terms of smallest tumor volume and insignificant variability in the body weight compared to the control groups (0.9% NaCl, Cur, MTX/Cur, M-Cur, and MTX-amide-M-Cur) (Fig. 9). These results evidenced the significance of imine linkage for the site-specific release of chemotherapeutic payload as well as a dual role of MTX as chemotherapeutic agent and targeting ligand for FA-receptors overexpressed on the surface of BC cells [147].

A Chinese patent has also been granted on Cur-loaded PMs (triphenylphosphine-chitosan-stearic acid graft) prepared by ultrasonication technique and evaluated for the management of BC (MCF-7 cells) [148]. The developed PMs exhibited ultra-fine particle size, high encapsulation efficiency, good percent yield, and protection of Cur against the photo-oxidation. The incubation of Cur-loaded PMs with the MCF-7 cells displayed a significantly higher cell uptake efficiency, cytotoxicity, and inhibited migration and invasion compared to the free Cur [148].

### Niosomes

Niosomes are innovatively designed nanovesicles that have been extensively developed for targeted delivery of chemotherapeutics [149]. These vesicular delivery systems can overcome different problems associated with bioactive(s) such as poor aqueous solubility, low oral bioavailability, degradation, and low endocytosis into various cells including the BC cells [150]. These nanovesicles are formed by the combination of cholesterol and non-ionic surfactants.

Owing to their unique composition and structural simulation with the biological membrane, niosomes have been extensively deployed as a drug delivery carrier for a wide variety of drugs to improve their physicochemical properties and biomedical efficacy [151]. One of the promising features of the niosomes is ability of simultaneous delivery of multiple payloads with different physicochemical properties (such as co-delivery of hydrophilic and hydrophobic drugs) [152]. Naderinezhad et al. [152] designed a hybrid model of niosomes by combining the liposomes and niosomes (LipoNiosome) together for co-delivery of Dox and Cur and evaluated for anticancer efficacy against the various cancer cells. The engineered Cur-Dox-LipoNiosomes exhibited an ultra-small particle size (⁓40 nm), high encapsulation efficiency (⁓80%), pH-responsive release of entrapped drugs (predominant release at acidic pH compared to the physiologic pH), and sustained release kinetics. The cell uptake study suggested that LipoNiosome exhibited an excellent cell internalization ability with a specific uptake and higher cytotoxicity in all cancer cells compared to the free drugs [152].

Aiming to extend the plasma circulation time and improving the passive transfection into BC cells (MCF-7), Alemi and co-researchers [153] developed PEG-functionalized Cur/PTX co-loaded niosomes. The PEGylated niosomes (PEG-Cur/PTX-niosomes) exhibited an ultrafine vesicle size (~ 90 nm), excellent encapsulation efficiency (~ 100%), good zeta potential (+ 15 mV), and satisfactory colloidal stability. The PEG-Cur/PTX-niosomes showed the pH-responsive release with predominant release at an acidic pH and relatively a slower release at the physiologic pH which indicate their ability to specifically release the therapeutic cargo at TME. The cell uptake study demonstrated a specific uptake of PEG-Cur/PTX-niosomes into MCF-7 cells (BC cells) compared to the MCF-10A (normal epithelial breast cells). The cytotoxicity study suggested a significantly higher cytotoxicity in MCF-7 cells treated with PEG-Cur/PTX-niosomes compared to the unPEGylated Cur/PTX-niosomes and the free drugs [153].

Another novel functionalization strategy was proposed by Akbarzadeh et al. [154] in which they designed Cur-loaded niosomes shelled with calcium alginate (CA) and evaluated for the treatment of BC. Due to unique properties of CA, these delivery systems were expected to have better biopharmaceutical and therapeutic potential. The anticancer efficacy of CA-shelled Cur-niosomes was evaluated against various BC cells (SKBR3 and MDA-MB231) and results were compared with the normal breast cells (MCF-10A cells). The fabricated CA-Cur-niosomes exhibited nano-scaled size, good encapsulation efficiency, and good colloidal stability (up to 1 month at 4 °C). The release study depicted a pH-responsive release behavior of CA-Cur-niosomes with predominant release at acidic pH and relatively lower release at physiological pH [154]. In addition, the developed CA-Cur-niosomes showed good biocompatibility against the MCF10A cells but higher cytotoxicity against SKBR3 and MDA-MB231 cells. The cell uptake study indicated a specific endocytosis of CA-Cur-niosomes into BC cells compared to relatively lower internalization into MCF-10A cells. The biochemical analysis revealed a significant suppression in the expression of cancerous genes (e.g., Bcl2, cyclin E and cyclin D) and higher expression of pro-apoptotic genes (e.g., caspase-3, Bax, P53 and caspase-9) in BC cells treated with CA-Cur-niosomes compared to unfunctionalized Cur-niosomes and the free Cur [154].

**Fig. 10:**
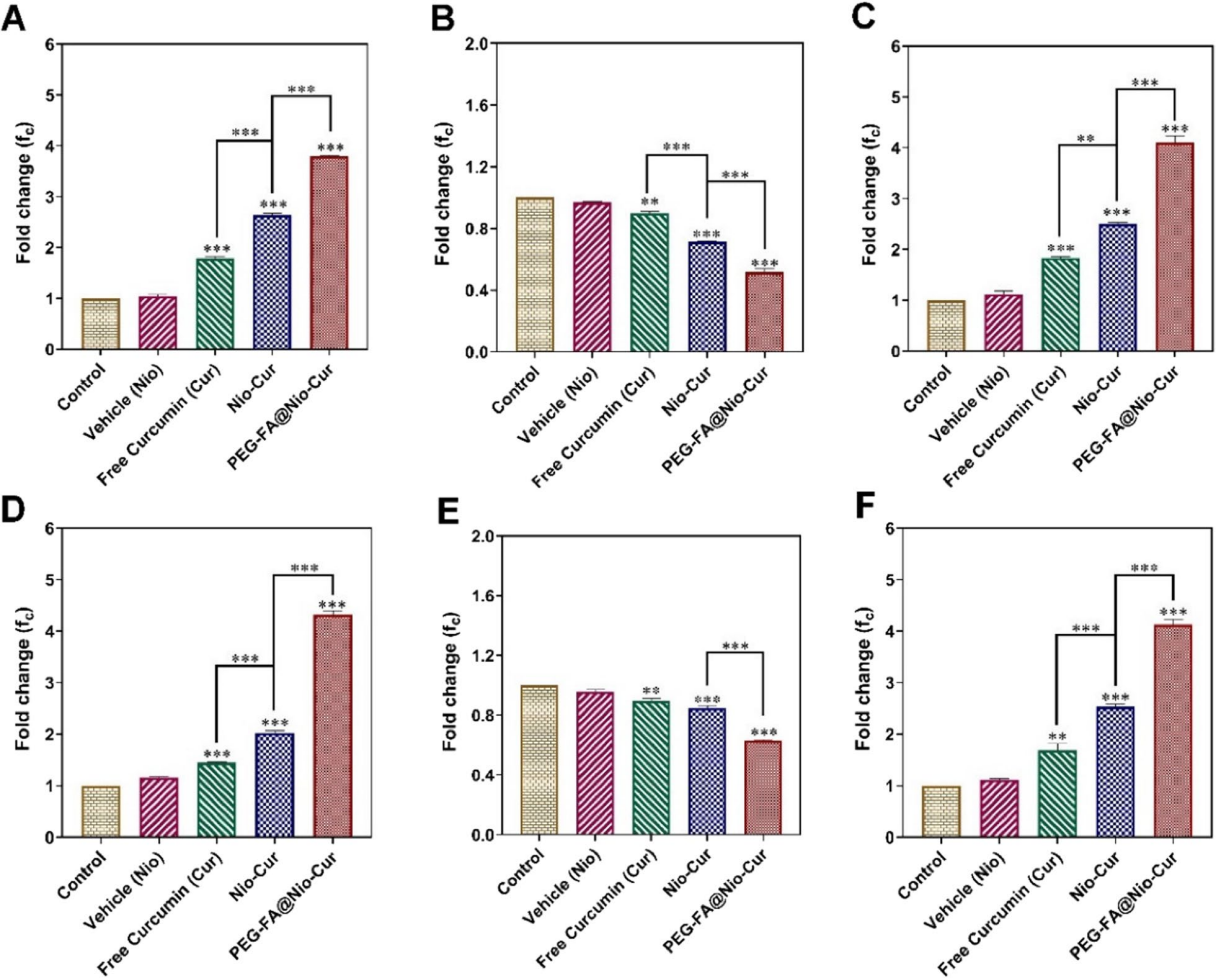
Upregulated expression of Bax (**A, D**) and p53 genes (**C, F**) and downregulation in the expression of Bcl2 (**B, E**) in MCF7 and 4T1 cells treated with PEG-FA@Nio-Cur compared to vehicle (Nio), free Cur, and unfunctionalized Nio-Cur. Adapted from [158]

Multifunctionalization is an emerging adaptation in the nanotechnology to achieve versatile benefits such as prolonging of plasma circulation time (via PEGylation), stimuli-responsive behavior (e.g., pH, temperature, light, etc.), specific targetability (via targeting ligand), simultaneous delivery of multi-therapeutics (e.g., genes, DNA, RNA, chemotherapeutics, proteins, etc.), and many more [155–157]. Keeping in view of the diversity of this relatively newer strategy of functionalization, Honarvari et al. [158] designed FA-decorated PEGylated Cur-niosomes (PEG-FA@Nio-CUR) aiming to prolong the plasma half-life, to achieve specific targetability, and to augment cell uptake efficiency via FA-receptor mediated endocytosis into BC cells. The developed PEG-FA@Nio-Cur exhibited nano-scaled vesicle size (⁓190 nm), narrow PDI, good zeta potential (−28 mV), good colloidal stability and high encapsulation efficiency (> 90%). The multifunctionalized PEG-FA@Nio-CUR also displayed a pH-responsive release behavior with predominantly higher release at acidic pH (pH 5.4) and relatively lower release at physiologic pH (pH 7.4) which indicates their target-specific release into TME. The cytotoxicity study showed highest cell viability in MCF-10A cells (normal breast epithelial cells) treated with PEG-FA@Nio-Cur which indicated good cytocompatability compared to unfunctionalized Nio-Cur and free Cur. The cell viability against the BC cells (MCF7 and 4T1) showed highest cytotoxicity (lowest IC_50_) of PEG-FA@Nio-Cur compared to the controls. Furthermore, a significant upregulation in the expression of Bax and p53 genes with marked downregulation in the expression of Bcl-2 were observed in MCF-7 and 4T1 cells treated with PEG-FA@Nio-Cur compared to other treatments (Fig. 10) [158]. The cell uptake study also indicated the dominance of PEG-FA@Nio-Cur for endocytosis into BC cells compared to unfunctionalized Nio-Cur and free Cur. These results concluded that multifunctionalization is a promising adaptation in the design of niosomes to improve selective targetability and anticancer efficacy against the BC [158]. Likewise, Lin et al. [159] developed FA-PEG-Cur-NLCs and evaluated for physicochemical characterization, pH-responsive release, stability, cytotoxicity, biocompatibility, cell uptake efficiency, specific internalization, and anti-tumor efficacy against the BC. The fabricated FA-PEG-Cur-NLCs exhibited ultrafine particle size (127 nm), cationic zeta potential (+ 13 mV), and high encapsulation efficiency (⁓85%). The drug release study depicted a relatively slower release of Cur from FA-PEG-Cur-NLCs compared to unfunctionalized NPs which indicated their sustained release behavior. The cytotoxicity study displayed a significantly higher (3.5-fold) toxicity in MCF-7 cells treated with FA-PEG-Cur-NLCs compared to unfunctionalized Cur-NLCs and the free Cur. The superior antitumor efficacy of FA-PEG-Cur-NLCs was also validated in animals (Balb/c nude mice) in terms of smallest tumor volume (312 mm^3^) compared to the control groups following the IV administration. These results demonstrated that functionalization of NPs is a promising strategy to augment anticancer efficacy of Cur [159]. The anti-tumorigenesis efficacy of FA-functionalized niosomes has also been recently validated by Rezaei et al. [160] for the treatment of BC. Results suggested a significantly higher cell uptake, cytotoxicity, and apoptosis in MCF-7 and 4T1 cells treated with FA-functionalized niosomes compared to unfunctionalized niosomes. Similar findings have also been reported by other studies [161–164].

### Dendrimers

Dendrimers are highly ordered, branched, and high molecular weight polymeric macromolecules which are formed of many functional groups. Due to their unique compact architecture, low polydispersity, and intrinsic physicochemical properties, dendrimers have gained remarkable recognition as drug delivery devices for a wide variety of drugs, proteins, genes, and RNAs. Unlike the traditional polymers, good aqueous solubility, polyvalency, biocompatibility, and precise molecular weight of dendrimers make them highly suitable drug delivery device for various biological applications [87, 165–167].

Keeping in view of their unique physicochemical features, Debnath and co-researchers [168] developed Cur-loaded dendrimers for improving the physicochemical properties and anticancer efficacy of Cur against the BC. A significant improvement in aqueous solubility, bioavailability, transfection efficiency, and cytotoxicity against the BC cells (SKBr3 and BT549 cell lines) was evident compared to the free Cur. The potent anticancer efficacy against the BC cells was attributed to an induction of apoptotic cell death via the activation of caspase-3 [168].

Aiming to confer an active targeting feature to the dendrimers, Shi et al. [169] developed Cur-loaded PAMAM dendrimers and functionalized with FA. The fabricated FA-Cur-dendrimers were evaluated for various physicochemical properties, pH-responsive release, and specific cell uptake efficiency into BC cells. A significant improvement in the aqueous solubility of Cur was evident from FA-Cur-dendrimers. The release study depicted a pH-responsive release of Cur with predominant release at an acidic pH which was attributed to presence of acid-labile phenolic ester group [169]. The cell uptake study showed a significant upregulation in the transfection efficiency of Cur into the BC cells from FA-Cur-dendrimers compared to unfunctionalized Cur-dendrimers and free Cur, and it was expected to be due to FA-receptor mediated endocytosis. These results evidenced that functionalization is a promising adaptation in the design of dendrimers to maximize the specific targetability and anticancer efficacy of Cur-nanomedicines against the BC [169].

### Other nanosystems

**Fig. 11:**
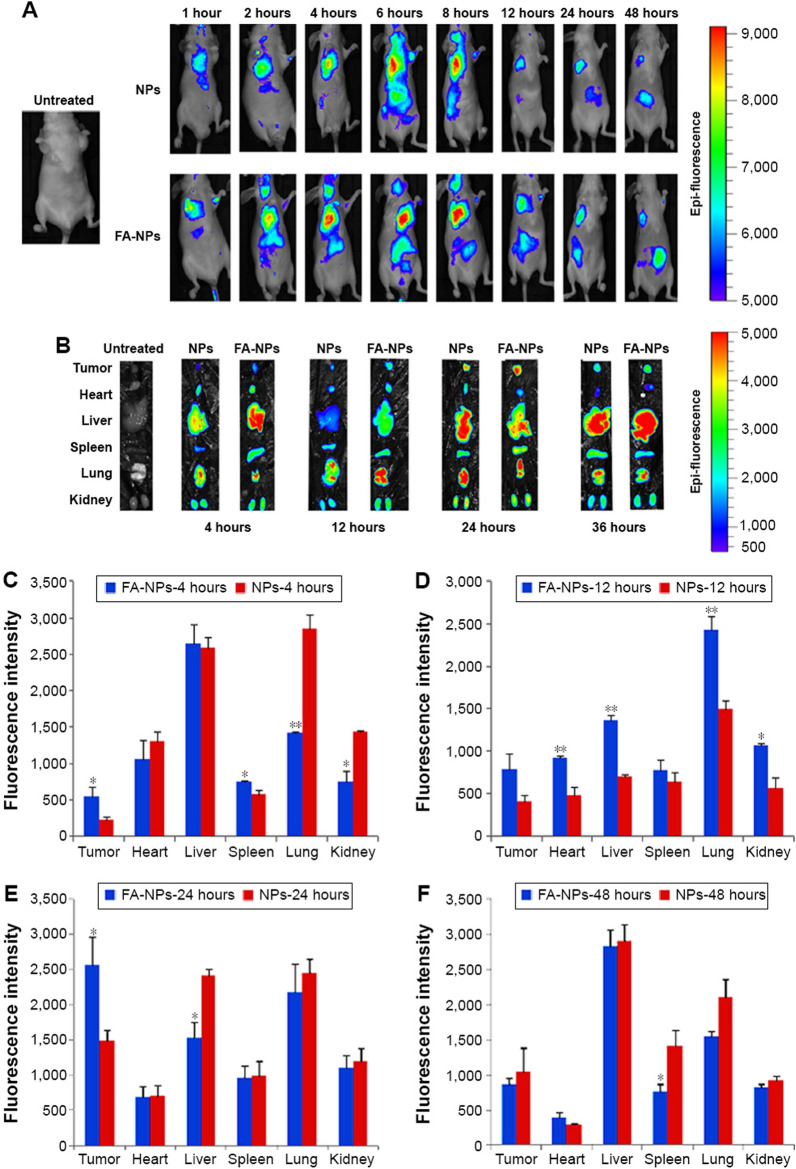
In vivo  biodistribution of FA–PEG–TiO_2_–NPs into tumor tissues and other body organs compared to unfunctionalized TiO_2_-NPs after injection into tail vein (**A**), ex vivo fluorescence imaging of dissected organs (**B**), and fluorescence intensity in different organs and tumor at 4 (**C**), 12 (**D**), 24 (**E**) and 48 hours (**F**). Adapted from [170]

A variety of other nanosystems have also been engineered for improving the pharmacokinetic profile, cell uptake efficiency, specific targetability, and cytotoxicity of Cur against the BC. Keeping in view of unique physicochemical properties, inorganic architecture, morphological features, surface characteristics, ultrafine particle size, and high stability, Ding et al. [170] developed TiO_2_-NPs for the co-delivery of Salvianolic acid B (Sal B) and Cur to achieve synergistic anticancer efficacy against the BC. Sal B is a traditional herb extensively used in the China as a circulation enhancing agent. In addition to its potent antioxidant, anti-inflammatory, and anti-coagulation effect, Sal B possess a moderate anticancer efficacy against a variety of cancers including the BC. Therefore, Ding and co-workers proposed the co-encapsulation of Cur and Sal B into the TiO_2_-NPs for a synergistic anticancer efficacy against the BC. Furthermore, to prolong the plasma circulation time and to achieve the specific targeting into the BC cells, the fabricated NPs were functionalized with PEG and FA (as targeting ligand). The fabricated FA-PEG-Cur/SalB-TiO_2_-NPs exhibited an ultrafine particle size (15–26 nm), narrow PDI, and good loading capacity. The cell cytotoxicity experiment showed a dose-dependent and significantly higher cytotoxicity in MCF-7 and MDA-MB-231 cells treated with FA-PEG-Cur/SalB-TiO_2_-NPs compared to unfunctionalized Cur/SalB-TiO_2_-NPs and free drugs (Cur and Sal B). The superior anti-proliferative efficacy of FA-PEG-Cur/SalB-TiO_2_-NPs was attributed to their highest uptake into both BC cells (MCF-7 and MDA-MB-231) compared to unfunctionalized Cur/SalB-TiO_2_-NPs and free drugs. The anticancer efficacy of FA-PEG-Cur/SalB-TiO_2_-NPs was also tested *in vivo*. The experimental animals (MDA-MB-231 bearing nude Balb/c mice) were injected (into tail vein) the test formulations and evaluated for biodistribution of Cur and Sal B into various tissues including the tumor tissues. The resulting fluorescent images clearly evidenced a time-mannered biodistribution of FA-PEG-TiO_2_-NPs into the tumor and liver tissues with subsequent excretion from the body (Fig. 11) [170]. Initially at 4 hours post-injection, the biodistribution was mainly observed in the liver tissues which was subsided at 12 h and become equivalent to the tumor tissues. After 24 h, the biodistribution was highest in the tumor tissues with subsequent decrease in 48 h which indicate a successful excretion of drugs from the body. These results evident the biopharmaceutical and therapeutic feasibility of inorganic NPs (TiO_2_-NPs) for the successful co-delivery of Cur and Sal B for the treatment of the BC [170].

An innovative design of magnetic NPs (Fe_2_O_3_-NPs) was also granted US-patent in 2013 in which β-cyclodextrin (β-CD)-citrate coated Fe_2_O_3_-NPs were prepared and loaded with Cur. The FTIR, XRD, and ^1^H NMR data indicated the formation of an inclusion complex of Cur into β-CD which resulted in a sustained release of Cur from the inclusion complex [171]. The physicochemical characterization of synthesized Cur-loaded β-CD-citrated coated Fe_2_O_3_-NPs showed nanoscaled dimension (<10 nm), high anionic surface charge (20–35 mV), good encapsulation efficiency, and good aqueous solubility (⁓60 mg/mL). Authors of this work demonstrated that these magnetic NPs can be used as a contrast agent as well as therapeutic agent against the BC which make them a successful theranostics for early detection and treatment of BC [171]. A summary of different types of functionalization strategies (e.g., PEGylation, targeting ligand conjugation, stimuli-responsive release, multifunctionalization, etc.) adapted for the improvement of pharmacokinetic profile, selective targeting, and antitumor efficacy of Cur against the BC has been presented in the Table 2.

**Table 2:** List of different functionalizations adapted for various Cur-nanomedicines for the treatment of BC

Type of nanosystem	Type of functionalization	BC model	Study type (in vitro/in vivo)	Study parameters	Major findings	References
Liposomes	Lecithin (fatty acid)	MCF7 (human BC cells with overexpressed estrogen, progesterone or glucocorticoid receptors)	In vitro	Cell compatibility Cytotoxicity Anticancer efficacy	1. Dose dependent cytotoxicity was observed in MCF-7 cells incubated with CUR-liposomes compared to free Cur 2. Good compatibility with no signs of toxicity observed in MCF-10A cells (normal breast cells) compared to free Cur 3. Liposomes composed of salmon lecithin produce better cytotoxicity in MCF7 cells compared to soya and rapeseed lecithins 4. Salmon lecithin functionalized liposomes produces significantly higher cytotoxicity in MCF7 cells due to enhanced ROS generation compared to free Cur	[72]
	FA functionalization	MCF-12A (non-malignant BC cells), MDA-MB-231 MDA-MB-231 (triple negative BC cells	In vitro	Specific targetability via overexpressed FA receptors LD_50_ Antitumor efficacy	1. MDA-MB-231 treated with folate tagged CUR-liposomes showed higher uptake 2. LD_50_ of folate tagged CUR-liposomes in MDA-MB-231 was significantly lower (19 µM) than normal breast cells (MCF-12A) which evidence specificity of liposomes against malignant BC cells	[77, 78]
	FA functionalization	MCF-7 cells	In vitro	Cell uptake Cytotoxicity Induction of apoptosis Anticancer efficacy	1. Significant increase in cell uptake, cytotoxicity, and anticancer efficacy compared to unmodified Cur-loaded liposomes and free Cur	[79]
	Affibody functionalization	SKBR3 (HER2 overexpressed BC cells) and MCF-7	In vitro	Cell uptake Cytotoxicity Induction of apoptosis Anticancer efficacy	1. Significant increase in cytotoxicity, cell uptake, and anticancer efficacy compared to unmodified Cur-loaded liposomes and free Cur	[80]
	PEGylation	MCF7 and B16F10 cells	In vitro	Cell uptake Cytotoxicity Cell migration IC_50_ Anticancer efficacy	1. PEGylated liposomes exhibited significantly high cell uptake efficiency 2. Improved cytotoxicity with low IC_50_ value and compared to unfunctionalized liposomes 3. Cell migration was significantly reduced which indicates higher anti-metastatic efficacy	[82]
Solid Lipid Nanoparticles (SLNs)	Steric acid and lecithin	SKBR3 cells	In vitro	IC_50_ Cell uptake Induction of apoptosis Anti-proliferative efficacy	1. IC_50_ (18.78 µM) of CUR-SLNs was significantly lower than free CUR (28.42 µM) 2. Significantly higher cell uptake was observed in CUR-SLNs in comparison with free Cur 3. Higher cytotoxicity was observed in SKBR3 BC cells in comparison with free drug 4. Higher induction of apoptosis via generation of reactive oxygen species (ROS) in comparison compared with free drug	[86]
	Transferrin functionalization	MCF-7 cells	In vitro	Release profile CUR protection from photo-degradation Cell uptake Cytotoxicity	1. Sustained release profile 2. Good protection of CUR against auto-oxidation and photo-degradation 3. Significant improvement in cell uptake and cytotoxicity compared to free CUR and unmodified CUR-SLNs	[97]
	PEGylation + FA functionalization	MCF-7 and MDA-MB-231 cells	In vitro	Cell uptake Targeted biodistribution Cytotoxicity	1. Functionalized SLNs exhibited significantly higher uptake of Cur and DOX 2. Targeted biodistribution of Cur and DOX into BC cells 3. Synergistic antitumor efficacy with enhanced cytotoxicity compared to both drugs alone	[98]
Albumin NPs	Albumin functionalization	MDA-MB-231	In vitro & In vivo	Release profile Cell uptake Cytotoxicity Pharmacokinetic profile Anticancer efficacy	1. Sustained release of encapsulated CUR up to one month 2. Higher cytotoxicity and passive accumulation into BC cells compared to free CUR 3. Improved absorption, bioavailability, and biodistribution into cancer cells	[100]
	PDL-1 (programmed death ligand-1) functionalization	MCF7 and MDA-MB-231 cells	In vitro	Dose dependent cytotoxicity IC_50_ Cell uptake Fluorescence	1. Dose-dependent and time mannered increase in cytotoxicity in BC cells 2. IC_50_ of PDL-1-conjugated CUR-NPs (61 μM) against MDA-MB-231 cells was 4 times less than IC_50_ of free CUR (234 μM) 3. Higher fluorescence with PDL-1 conjugated albumin NPs compared to free CUR and unmodified albumin NPs	[101]
	PEGylation	MDA-MB-231 cells	In vitro	Release profile Cell uptake Stealthing effect Cytotoxicity	1. Biphasic release with quick release followed by sustained release over 35 days 2. Increased cell uptake into BC cells compared to free CUR 3. Low uptake into kupffer and liver cells compared to free Cur. It indicates good stealthing effect of PEG 4. Higher cytotoxicity compared to non-PEGylated NPs and free Cur	[102]
	Multi-functionalization (citrate functionalized albumin coated Cur/5-flurouracil co-loaded FeO_3_-NPs + FA conjugation	MCF-7	In vitro	Cell uptake Cytotoxicity Specific internalization	1. Significant increase in cell uptake efficiency compared to unfunctionalized NPs and free drugs 2. Synergistic cytotoxicity compared to unfunctionalized NPs and free drugs	[103]
Polymeric NPs	PLGA NPs functionalized with TPGS, PEG, dextran or chitosan	MCF-7 cells	In vitro	Cell uptake Cell cycle termination Antioxidant efficacy IC_50_ Cytotoxicity	1. Capping with different capping agents enhanced cell uptake efficiency of Cur and DTX compared to unconjugated PLGA NPs 2. Highest cell uptake obtained with TPGS-capped PLGA NPs 3. Termination of cell cycle at G2/M phase 4. IC_50_ was lowest in case of TPGS-capped Cur/DTX co-loaded PLGA NPs compared to unfunctionalized PLGA NPs and free drugs 5. Significantly higher cytotoxicity with TPGS-capped PLGA NPs compared to other capping agents and free CUR	[117]
	PEGylation	MDA-MB-231 cells	In vitro & In vivo	Cell uptake IC_50_ Cytotoxicity via apoptosis Cell viability Plasma circulation time and bioavailability Metastasis and angiogenesis	1. Significantly higher uptake in BC cells compared to unmodified PLGA NPs and free Cur 2. Enhanced cytotoxicity via induction of apoptosis compared to free CUR and unmodified PLGA NPs 3. Lowest IC50 compared to control 4. Suppression of proliferation in BC cells 5. Significantly higher suppression of invasion (MMP-9) and angiogenesis (VEGF) compared to free CUR and unmodified PLGA NPs 6. Higher bioavailability compared to free CUR	[122]
	Lipid coating	HUVECs (cells derived from endothelial of vein from human umbilical cord) and MDA-MB-231 cells	In vitro	Cell proliferation Cytotoxicity via apoptosis Cell viability Metastasis and angiogenesis	1. Dose- and time-dependent cytotoxicity which was significantly higher (lower IC_50_) than the free CUR 2. Adhesion of CTCs to the endothelial cells (a hallmark of cancer metastasis) was significantly decreased after treatment of lipid-coated CUR-loaded PLGA NPs compared to free CUR 3. Suppression of proliferation and metastasis of cancer cells	[123]
	Transferrin or anti-TAG-72 monoclonal antibody (Mab) functionalization	MDA-MB-231 cells	In vitro	Release kinetics Cell proliferation Colony formation Cytotoxicity via apoptosis	1. Sustained release over 25 days 2. Significant improvement of specific cell uptake into BC cells compared to unfunctionalized NPs and free Cur 3. Downregulated proliferation and colony formation compared unfunctionalized NPs and free Cur 4. Significant increase in cytotoxicity via induction of apoptosis	[134]
Polymeric micelles (PMs)	Hyaluronic acid (HA) functionalization	4T1 cells bearing mice (4T1 cells are derived from mammary gland tissues of BALB/C mice)	In vitro and In vivo	Release at different pH Tumor growth Cardiotoxicity and hepatotoxicity	1. Predominant release of drugs at acidic pH (4.5) (simulating with tumor microenvironment) compared to alkaline pH (7.4) 2. Significantly suppressed tumor growth (55%) in mice treated with HA-functionalized Cur/DOX co-loaded PMs compared to unfunctionalized PMs and free drugs (27%) 3. Better compatibility with no signs of cardiotoxicity and hepatotoxicity in contrast to free doxorubicin	[142]
	Cyclin-D1 functionalization	MCF-7 cells	In vitro	Cell proliferation Tumor invasion and metastasis Oral bioavailability Cyclin-D1 expression	1. Significantly higher suppression of BC cell proliferation (83.6%) compared to commonly used chemotherapeutic agents 2. Significantly higher oral bioavailability compared to free CUR 3. Cyclin-D1 expression was remarkably decreased compared to other chemotherapeutic agent, indicating specific saturation and targeting	[143]
	Methotrexate (MTX) functionalization	MCF-7 and HeLa tumor-bearing BALB/c nude mice	In vitro & In vivo	pH-responsive release Cell uptake Cytotoxicity Anticancer efficacy	1. Release of Cur was pronounced at acidic pH (pH 5.0) compared to physiologic pH (7.4) indicating tumor-specific release 2. Higher cell uptake into MCF7 compared to unfunctionalized PMs and free Cur 3. Following IV administration, significant reduction in tumor volume compared to controls	[147]
Niosomes	Hybrid niosomes (LipoNiosome prepared by merging of niosome & liposome)	SKBR3 and MDA-MB231 cells	In vitro	Release profile at different pH Cell proliferation Cell uptake Cytotoxicity via induction of apoptosis Gene expression	1. Predominant release at acidic pH (simulating with tumor microenvironment) compared to alkaline pH 2. Higher uptake into BC cells compared to conventional liposomes and individual free drugs (Cur & DOX) 3. Synergistic inhibition of cell proliferation compared to conventional liposomes and individual free drugs (Cur & DOX) 4. Higher cytotoxicity due to induction of apoptosis	[152]
	PEGylation	MCF7	In vitro	Release profile at different pH Cell proliferation Cell uptake Cytotoxicity	1. Predominant release at acidic pH (simulating with tumor microenvironment) compared to alkaline pH 2. Higher uptake into BC cells compared to normal epithelial breast cells (MCF-10A) 3. Synergistic cytotoxicity compared to individual free drugs (Cur & PTX) 4. Higher cytotoxicity due to induction of apoptosis	[153]
	Calcium alginate shell functionalization	SKBR3, MDA-MB231, and MCF10A cells	In vitro	Release profile at different pH Cell proliferation Cell uptake Cytotoxicity and mechanism	1. Predominant release at acidic pH (simulating with tumor microenvironment) 2. Higher uptake into cancer cells compared to free drug 3. Good biocompatibility against normal epithelia BC cells compared to free Cur 4. Higher cytotoxicity in BC cells compared to free Cur, due to upregulation of pro-apoptotic genes and downregulation of cancerous genes	[154]
	PEGylation + FA functionalization	MCF-7, 4T1, and MCF10A cells	In vitro	Release profile at different pH Cytocompatability Cell proliferation Cell uptake Cytotoxicity IC_50_	1. Predominant release at acidic pH (simulating with tumor microenvironment) (pH 5.4) compared to physiologic pH (7.4) 2. Good compatibility against MCF10A cells compared to free Cur 3. Higher uptake into BC cells compared to free Cur 4. Higher cytotoxicity in BC cells with lower IC_50_ value compared to free drugs 5. Significant upregulation in the expression of Bax and p53 genes with marked downregulation in the expression of Bcl-2 in MCF7 and 4T1 cells	[158]
	PEGylation + FA functionalization	MCF-7 and MCF10A cells	In vitro & in vivo	Release profile at different pH Cytocompatability Cell proliferation Cell uptake Cytotoxicity IC_50_ Tumor grwoth	1. Predominant release at acidic pH (simulating with tumor microenvironment) (pH 5.4) compared to physiologic pH (7.4) 2. Good compatibility against MCF10A cells compared to free Cur 3. Higher uptake into BC cells compared to free Cur 4. Higher cytotoxicity in BC cells with lower IC_50_ value compared to free drugs 5. Significant reduction in tumor growth in animals treated with FA-PEG-CUR-NLCs compared to unfunctionalized NLCs and free Cur	[159]
Dendrimer	FA functionalization	MDA-MB 231 cells	In vitro	Aqueous solubility Release profile at different pH Transfection efficiency Permeability and retention Cytotoxicity	1. Significant improvement in aqueous solubility of CUR and PTX after encapsulation into dendrimers 2. Predominant release at acidic pH (simulating with tumor microenvironment) 3. FA functionalization significantly improved transfection efficiency into BC cells compared to free drugs 4. Enhanced Permeability and Retention (EPR) effect 5. Potent anticancer agent against BC cells	[169]
Inorganic NPs (TiO_2_-NPs)	PEGylation + FA functionalization	MCF7 and MDA-MB-231 cells	In vitro & in vivo (MDA-MB-231 bearing nude Balb/c mice	Cell uptake Cytotoxicity Drug biodistribution	1. Moderate improvement in cell uptake efficiency of Cur into BC cells compared to unfunctionalized Cur/SalB-TiO_2_-NPs and free drugs 2. Dose-dependent synergistic cytotoxicity in BC cells treated with FA-PEG-Cur/SalB-TiO_2_-NPs compared to unfunctionalized Cur/SalB-TiO_2_-NPs and free drugs 3. Time-mannered biodistribution of FA-PEG-TiO_2_-NPs into tumor and liver tissues with subsequent excretion from the body	[170]

## Summary and conclusions

Despite exhibiting the promising anticancer efficacy against the BC, clinical translation of curcumin (Cur) is restricted due to chemical instability (e.g., photodegradation), hydrophobicity, poor absorption, low bioavailability, short plasma half-life, and lower distribution to different body tissues. To mitigate these shortcomings, diverse types of nanocarriers such as liposomes, SLNs, polymeric micelles, polymeric nanoparticles, niosomes, dendrimers, and inorganic NPs have been deployed as delivery devices for the Cur. The implication of nanotechnology has significantly augmented the physicochemical properties, bioavailability, plasma half-life, transfection efficiency, cell uptake, and anticancer efficacy against the BC. However, majority of the Cur-nanomedicines are still facing grander challenges due to recognition by the reticuloendothelial system, non-specific accumulation into various body tissues, poor penetration and accumulation in the TME, and multidrug resistance due to overexpressed P-glycoproteins in the tumor cells which ultimately hampers the clinical significance of Cur-nanomedicines.

In the recent decades, plentiful newest developments have been adapted to overcome challenges associated with Cur-nanomedicines and to augment their anticancer efficacy against the BC. Among these developments, the functionalization of Cur-nanomedicines has gained remarkable recognition. Many dynamic functionalization strategies have been adapted in the design of Cur-nanomedicines including the PEGylation, conjugation of targeting ligand(s), pH-responsiveness, co-delivery of multiple therapeutics, and multifunctionalization. The critical analysis of available literature revealed that PEGylation (physical decoration or chemical conjugation of PEG on NPs surfaces) can successfully extends the plasma half-life by repressing the recognition and subsequent metabolism of Cur-nanomedicines by the RES. Plethora of studies have reported that PEGylation significantly improves the passive uptake and internalization of Cur-nanocarriers into the BC cells with only minimal-to-no uptake into the normal epithelial breast cells. To maximize the specific uptake into the BC cells/tissues as well as to avert non-target accumulation, many researchers have adapted active targeting strategy in which Cur-nanomedicines were conjugated with diverse targeting ligands (e.g., FA, transferrin, PDL-1, hyaluronic acid, monoclonal antibody, affibody, etc.). The selection of a specific targeting ligand(s) to conjugate on the exterior of the Cur-nanomedicines mainly depends upon the type of biochemical target (e.g., FA-, transferrin- and/or CD44-receptors, or other genes and proteins) overexpressed on the surface of BC cells. The Cur-nanomedicines having capped with targeting ligand(s) resulted in a significant increase in specific uptake and internalization into BC cells which ultimately results in a significant decrease in IC_50,_ and enhanced cytotoxicity compared to unfunctionalized Cur-nanomedicines and the free drug(s). On the other hand, the Cur-nanomedicines exhibiting a pH-responsive behavior expressed predominant release at the acid pH (4.5–5.0) compared to the physiologic pH, which indicates their site-specific release at TME. Besides these functionalization, multifunctionalization is the most recent adaptation in the design of Cur-nanomedicines in which nanocarrier’ architecture is functionalized with multiple functionalities such as PEGylation + targeting ligand + pH-responsiveness + combo delivery of multiple therapeutics. These multifunctional Cur-nanomedicines displayed an exceptional potency and powerful anticancer efficacy against the BC while ensuring an immense safety against normal healthy cells. The convincing evidences compiled in this review article have demonstrated that diverse types of functionalizations can be adapted while designing a novel Cur-nanomedicine to improve the physicochemical properties, pharmacokinetic profile, specific cell uptake, and anticancer efficacy of Cur against the BC. Despite all these evolutions, several aspects of Cur-nanomedicines are still debatable such as poor reproducibility during the manufacturing, poor in vitro–in vivo correlation, unexplained nanotoxicity due to unwanted interaction of nanomaterials with the biological tissues, lacking the establishment of acute and chronic toxicities, and absence of specific international guidelines for the manufacturing, administration, and the safety of Cur-nanomedicines for the treatment of BC.

## Data Availability

Not applicable.
